# Multiphasic CT radiomics–clinical model for predicting acute pancreatitis recurrence: a multicenter survival analysis

**DOI:** 10.1186/s13244-026-02439-6

**Published:** 2026-11-04

**Authors:** Weixia Wu, Chang Rong, Shaoqi Wang, Yu Zhang, Xu Lin, Xiaoying Zhao, Xiaomin Zheng, Yichao Liu, Xingwang Wu

**Affiliations:** 1 Department of Radiology, The First Affiliated Hospital of Anhui Medical University, Hefei, China; 2 Department of Radiology, Bozhou People’s Hospital, Bozhou, China; 3 Department of Radiology, Lu’an People’s Hospital of Anhui Province, Lu’an, China

**Keywords:** Acute pancreatitis, Recurrence, Computed tomography, Radiomics, Risk assessment

## Abstract

**Objectives:**

To develop and externally validate a predictive model integrating multiphasic CT radiomics and clinical variables to estimate recurrence risk after first-episode acute pancreatitis (AP).

**Materials and methods:**

This multicenter retrospective study included 548 patients with first-episode AP (training, *n* = 272; internal validation, *n* = 117; external validation, *n* = 159). Clinical and multiphasic CT data were collected, with recurrence as the primary endpoint. After stability-based feature selection, clinical, radiomics, and combined Cox models were constructed. Performance was evaluated using the *C*-index and time-dependent AUC, with SHAP for interpretability.

**Results:**

A total of 548 patients were included (median age 42 years; 363 men). Recurrence rates were similar across cohorts (30.1%, 29.9%, and 28.3%; *p* = 0.702). The combined model achieved C-indices of 0.783 and 0.775 in the internal and external validation cohorts, respectively, numerically higher than those of the clinical model (0.748 and 0.750) and radiomics model (0.647 and 0.619), although the incremental improvement over the clinical model was not statistically significant. In the external validation cohort, time-dependent AUCs remained acceptable through 36 months but declined at 48 months. High-risk patients had lower 3-year recurrence-free survival than low-risk patients (37.4% vs 86.8%; log-rank *p* < 0.001). SHAP analysis identified the NC-3mm-Radscore as the most influential imaging-derived variable, whereas etiology-targeted prevention was the dominant protective factor.

**Conclusions:**

The combined model demonstrated acceptable discrimination for predicting recurrence within the first 24 months after first-episode AP and may facilitate individualized follow-up and secondary prevention strategies.

**Key Points:**

***Question*** Can a multiphasic CT radiomics–clinical model improve prediction of AP recurrence over clinical factors alone in a multicenter setting?

***Findings*** The combined model demonstrated numerically higher predictive performance than the clinical model, though the incremental improvement was not statistically significant.

***Critical relevance statement*** This study critically evaluates the added value of multiphasic radiomics for recurrence prediction in AP, demonstrating that while radiomics captures baseline microenvironmental vulnerability, post-discharge clinical interventions remain the dominant dynamic drivers.

**Graphical Abstract:**

**Figure:**
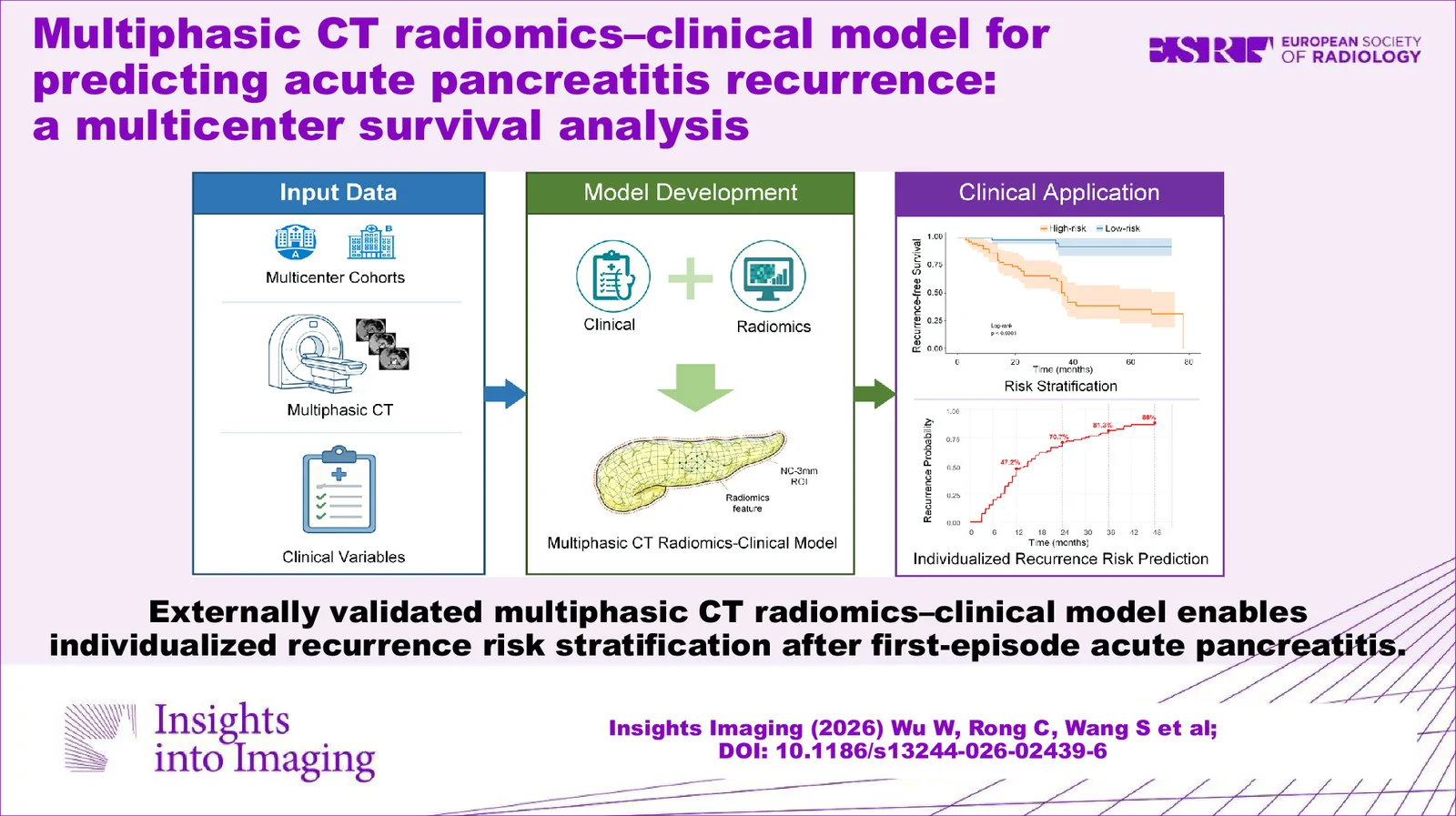

## Introduction

Acute pancreatitis (AP) is a common gastrointestinal emergency, and approximately 18–24% of patients develop recurrent acute pancreatitis (RAP) after a first episode [1, 2], substantially increasing the risk of chronic pancreatitis [3]. Early identification of patients at high risk of recurrence is therefore essential for individualized follow-up and secondary prevention [4, 5].

Current risk assessment for RAP relies predominantly on clinical variables and shows limited predictive performance [2, 6, 7]. Even in prospective multicenter studies, predictors remain largely restricted to conventional factors such as age and diabetes [2]. Although contrast-enhanced computed tomography (CECT) is routinely used for morphological assessment [8], its ability to predict long-term recurrence remains limited [9]. Importantly, accumulating evidence indicates that the peripancreatic microenvironment—particularly local lipolysis and fat necrosis—acts as a primary driver of inflammatory amplification in AP [10]. This pathophysiological mechanism suggests that evaluating structural alterations in the adjacent tissue may serve as a potential biomarker of recurrence susceptibility.

Radiomics enables high-throughput extraction of quantitative imaging features, capturing subtle tissue characteristics beyond conventional morphology [11–13]. However, existing studies are predominantly single-center, lack external validation, and use cross-sectional designs that do not account for time-to-event recurrence [12–14]. Reliance on single-phase imaging and variability in CT acquisition timing further limit model generalizability [15, 16].

To fill this gap, this multicenter study developed and externally validated a multiphasic CT radiomics–clinical model for survival-based prediction of recurrence after first-episode AP to improve individualized risk stratification.

## Materials and methods

### Study design and population

**Fig. 1:**
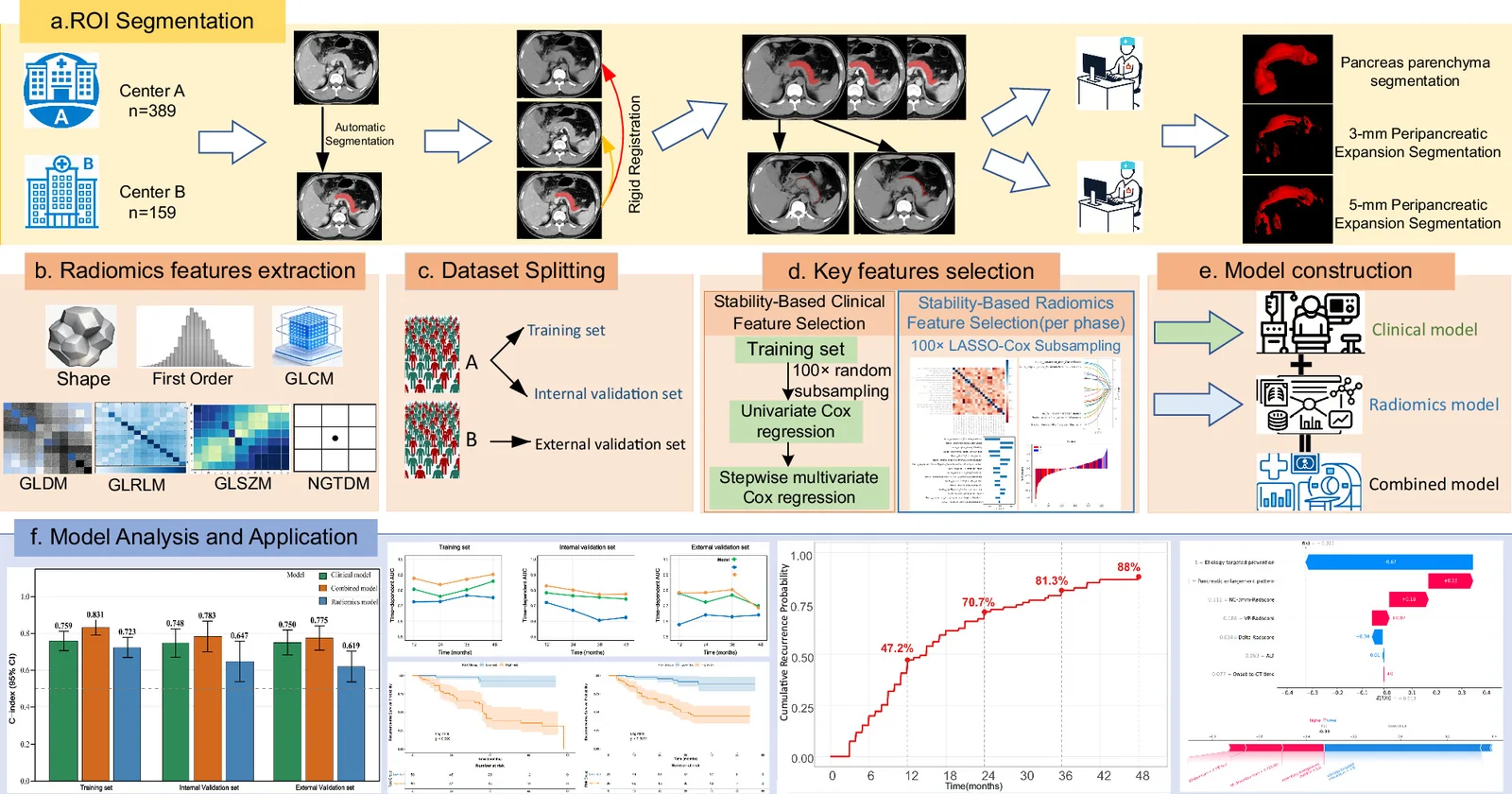
Flowchart of the overall study design. ROI, region of interest. **a** ROI segmentation from multiphasic CT images. **b** Radiomics feature extraction. **c** Dataset splitting into training and internal validation cohorts (Center A), and an external validation cohort (Center B). **d** Key feature selection using stability-based radiomics feature selection and repeated subsampling Cox regression for clinical variables. **e** Construction of the clinical, radiomics, and combined models. **f** Model analysis and application, including C-index evaluation, time-dependent AUC, Kaplan–Meier curves, individualized recurrence probability curves, and SHAP value analysis, among others

This retrospective multicenter study was approved by the institutional review boards of the participating centers, and the requirement for written informed consent was waived (The First Affiliated Hospital of Anhui Medical University, approval no. PJ202511-87; Bozhou People’s Hospital, approval no. 2025-248). Consecutive patients with first-episode AP admitted to two tertiary centers between November 2018 and October 2024 were included. The development cohort (Center A, *n* = 389) was randomly split into training and internal validation cohorts at a 7:3 ratio, and an independent external validation cohort comprised 159 patients from Center B. The study workflow is illustrated in Fig. 1.

**Fig. 2:**
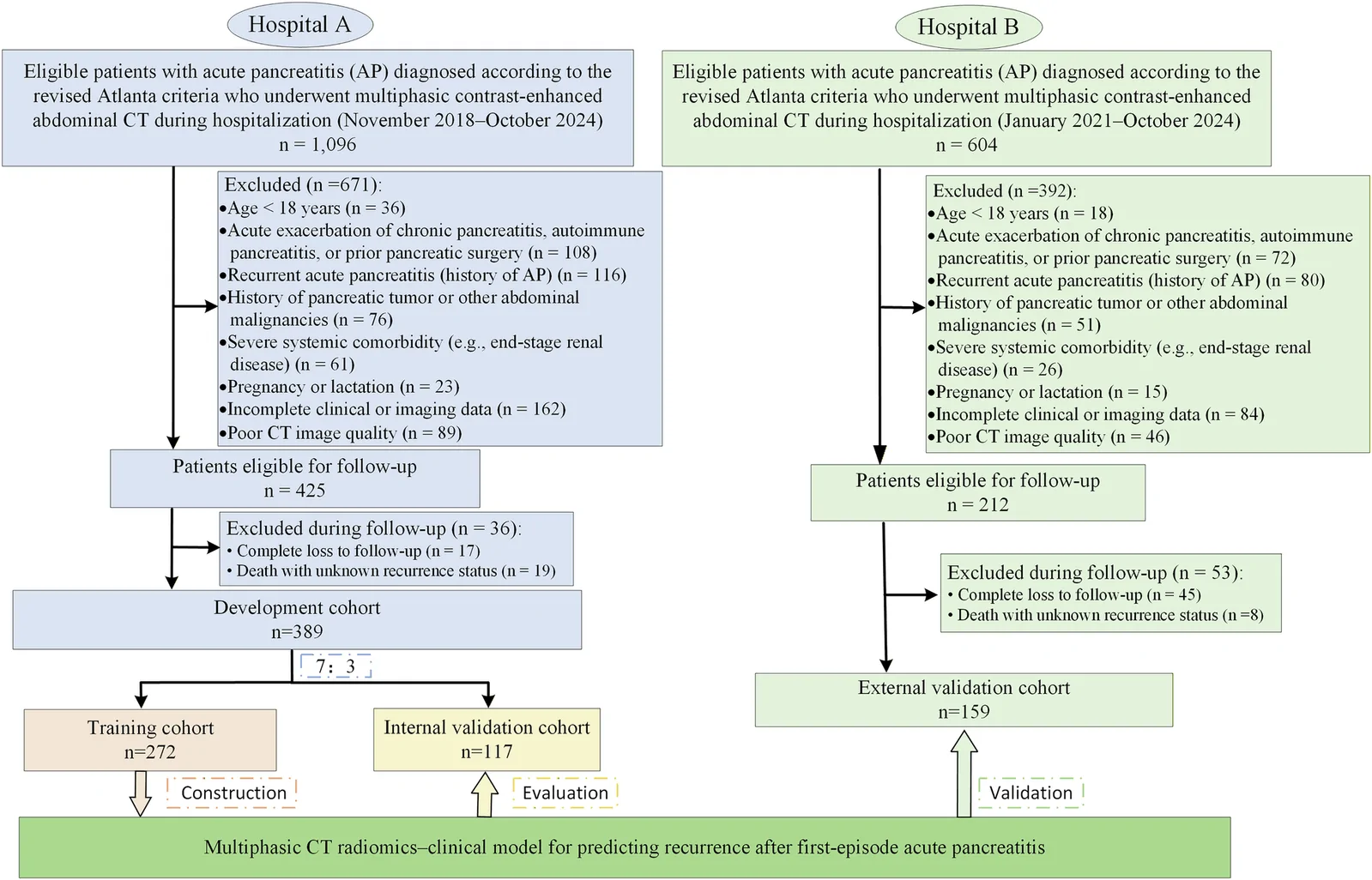
Flowchart of patient selection and cohort allocation

Eligible patients were adults with first-episode AP diagnosed according to the revised Atlanta classification [8] who underwent multiphasic CECT during hospitalization. Patients with chronic or autoimmune pancreatitis, recurrent AP, malignancy, prior pancreatic surgery, severe systemic comorbidities, incomplete data, poor image quality, or indeterminate recurrence status were excluded. The patient selection process is summarized in Fig. 2.

#### Clinical data and endpoints

Data included demographics, etiology, laboratory parameters (e.g., alanine aminotransferase [ALT], C-reactive protein [CRP]), imaging findings (including pancreatic morphology, pancreatic enlargement, pleural effusion, peritoneal effusion, and the modified CT severity index [mCTSI], which were independently assessed by two radiologists; Supplementary Method 1), and the interval from symptom onset to CT examination (onset-to-CT time). All patients received standard initial management (e.g., goal-directed fluid resuscitation, analgesia, early enteral nutrition) per international guidelines [17]. Following discharge, etiology-targeted secondary prevention (such as biliary intervention, lipid-lowering therapy, or alcohol cessation) was implemented and documented based on patient-specific risk factors.

The primary endpoint was RAP, defined according to the 2012 Atlanta criteria as a new episode occurring ≥ 3 months after discharge [3]. Follow-up via electronic medical records and telephone interviews until October 2025 was independently verified by two investigators.

Patients lost to follow-up immediately after discharge (*n* = 17) were excluded; otherwise, data were censored at the time of last contact.

#### CT image acquisition and evaluation

CT used 64-/128-slice scanners (GE, Philips, Siemens): 100–120 kV, 120–640 mA, 0.6–0.625 mm collimation, a 512 × 512 matrix; and 1–1.25 mm slice thickness reconstructed to 5 mm. CECT included arterial (20–30 s) and venous (60–70 s) phases.

### Image preprocessing and segmentation

For radiomics analysis, CT images were resampled to 1 × 1 × 1 mm³, followed by gray-level discretization (25-HU bin width), and *z*-score normalization (Supplementary Method 2). Regions of interest (ROIs) included the pancreatic parenchyma and peripancreatic regions expanded by 3 mm and 5 mm (NC-3mm and NC-5mm). These stepwise expansion boundaries were designed to quantify the spatial gradient of peripancreatic inflammation. As the immediate microenvironment is highly vulnerable to early microscopic exudation [10], this multi-scale approach ensures a more precise radiomic capture of localized inflammatory spread. Pancreatic segmentation was performed using nnU-Net v2 [18], which has been widely applied to abdominal organ segmentation [19–21]. Detailed methodology and performance results (cross-validation Dice: 0.853–0.873; external test Dice: 0.918) are provided in Supplementary Method 3 and Table S1.

### Radiomics feature extraction

Radiomics features were extracted from each imaging phase and ROI using the PyRadiomics engine, yielding 1874 features per phase-ROI combination (Supplementary Method 4).

### Feature selection and bias control

Feature selection was performed in the training cohort using a multistep stability selection strategy [22], including redundancy and correlation filtering, least absolute shrinkage and selection operator (LASSO)-Cox regression with selection-frequency filtering, and backward stepwise Cox regression. Phase- and region-specific Radscores were subsequently calculated from the selected features, of which NC-3mm-Radscore and VP-Radscore were retained due to stronger recurrence associations than the 5-mm region.

To capture dynamic enhancement effects, a Delta-Radscore (derived from relative feature changes between contrast-enhanced and non-contrast (NC) phases) was constructed using LASSO-Cox regression (Supplementary Method 5).

Clinical predictors were identified via repeated subsampling Cox regression, retaining features selected in > 80% of iterations. Onset-to-CT time was included to mitigate time-dependent bias [23] (Supplementary Method 6), and all model development steps—including feature selection, standardization, imputation, and model fitting—were confined to the training cohort.

### Model development, evaluation, and interpretation

Linearity was assessed via restricted cubic spline (RCS) (Fig. S1 and Supplementary Result 1). Three models were developed: clinical (demographics, labs, CT findings), radiomics (phase-/region-specific and Delta Radscores), and combined. Continuous variables were *z*-score standardized using training-cohort parameters.

Model comparison was performed using likelihood ratio tests and the Akaike information criterion (AIC). Model performance was evaluated using the concordance index (*C*-index), time-dependent area under the curve (AUC), calibration curves, Brier scores, and decision curve analysis (DCA). Incremental predictive value was assessed using integrated discrimination improvement (IDI) and continuous net reclassification improvement (NRI) (Supplementary Method 7).

Individual risk scores were calculated as weighted linear combinations of the selected predictors:

$${{\mathrm{Risk\; score}}}=\sum {{\upbeta }}_{{{{\rm{i}}}}}\times {X}_{{{{\rm{i}}}}}$$

where *X*ᵢ are the selected clinical and radiomics predictors, and βᵢ are the corresponding Cox regression coefficients estimated from the training cohort. Individual risk scores for validation were then calculated using the finalized regression coefficients from the combined model. The median training-cohort risk score was used as the cutoff for risk stratification, ensuring objectivity and reproducibility [24].

Model interpretability was assessed using Shapley additive explanations (SHAP) for global and local interpretations [25, 26]. The final model generated individualized cumulative recurrence probability curves (Supplementary Method 8).

### Statistical analysis

Categorical variables were compared using the χ² test or Fisher’s exact test, and continuous variables using the Student’s *t*-test or Mann–Whitney *U*-test, as appropriate. Variables with > 20% missing data were excluded. For the remaining variables (< 5% missingness), multiple imputation was performed using the *mice* package (*m* = 5) within the development cohort. In the external validation cohort, no key variables included in the final model had missing data; for other variables, single imputation was applied for descriptive analysis.

Risk stratification was evaluated using Kaplan–Meier analysis with the log-rank test. Statistical analyses were performed using R (version 4.5.2), and a two-sided *p* < 0.05 was considered statistically significant.

## Results

### Study cohort and patient baseline characteristics

A total of 548 patients with a first episode of AP were included, comprising a training cohort (*n* = 272; 82 recurrence events), an internal validation cohort (*n* = 117; 35 events), and an external validation cohort (*n* = 159; 45 events). Recurrence rates were comparable across cohorts (30.1%, 29.9%, and 28.3%, respectively; log-rank *p* = 0.702; Fig. S2). Baseline characteristics are summarized in Table S2. Most variables were well balanced, although significant differences were observed in several clinical and laboratory parameters. The interobserver agreement for CT feature assessment was good (κ = 0.80–0.90).

### Model construction and key feature selection

In addition to the mandatory onset-to-CT time covariate, the clinical model comprised three stable predictors: etiology-targeted prevention, pancreatic enlargement pattern, and ALT (Table S3).

For radiomics, NC-3mm-Radscore was retained as an independent predictor, whereas NC-5mm-Radscore was excluded. The final radiomics model included Delta-Radscore, VP-Radscore, and NC-3mm-Radscore (Table S4). The specific radiomics features constituting each Radscore, along with their selection frequencies and standardized coefficients, are detailed in Table S5. The combined model integrated both clinical and radiomic features, yielding seven predictors (Table 1). Among these, NC-3mm-Radscore (HR = 3.123) and Delta-Radscore (HR = 2.549) showed the strongest associations with recurrence risk. Although the pancreatic enlargement pattern was marginally significant (*p* = 0.061), it was retained based on model fit and selection stability (Supplementary Result 2 and Table S6).

**Table 1:** Multivariable Cox regression analysis of the combined model in the training cohort

Variable	β	HR	95% CI	*p*
Clinical factors				
Onset-to-CT time	0.134	1.144	0.986, 1.327	0.077
ALT	−0.714	0.490	0.275, 0.871	0.015
Pancreatic enlargement pattern	0.560	1.750	0.975, 3.141	0.061
Etiology-targeted prevention	−1.765	0.171	0.105, 0.281	< 0.001
Radiomics scores				
Delta-Radscore	0.936	2.549	1.694, 3.834	< 0.001
VP-Radscore	0.492	1.636	1.164, 2.300	0.005
NC-3mm-Radscore	1.139	3.123	1.524, 6.400	0.002

*HR* hazard ratio, *CI* confidence interval, *ALT* alanine aminotransferase

Delta-Radscore, the radiomics score derived from the percentage change between contrast-enhanced (arterial and portal venous phase) and NC radiomics features; VP-Radscore, the venous-phase radiomics score; and NC-3mm-Radscore, the radiomics score of the peripancreatic region extending 3 mm outward on NC CT. Variable coding was as follows: pancreatic enlargement pattern (1 = diffuse, 0 = focal) and etiology-targeted prevention (1 = yes, 0 = no)

### Risk score formulation

Risk score = (0.134 × z-onset-to-CT time)-(0.714 × z-ALT) - (1.765 × etiology-targeted prevention) + (0.560 × pancreatic enlargement pattern) + (0.936 × Delta-Radscore) + (0.492 × VP-Radscore)+(1.139 × NC-3mm-Radscore).

Continuous variables were standardized (*z*-scores), and categorical variables were coded as defined in Table 1.

### Discrimination performance

**Fig. 3:**
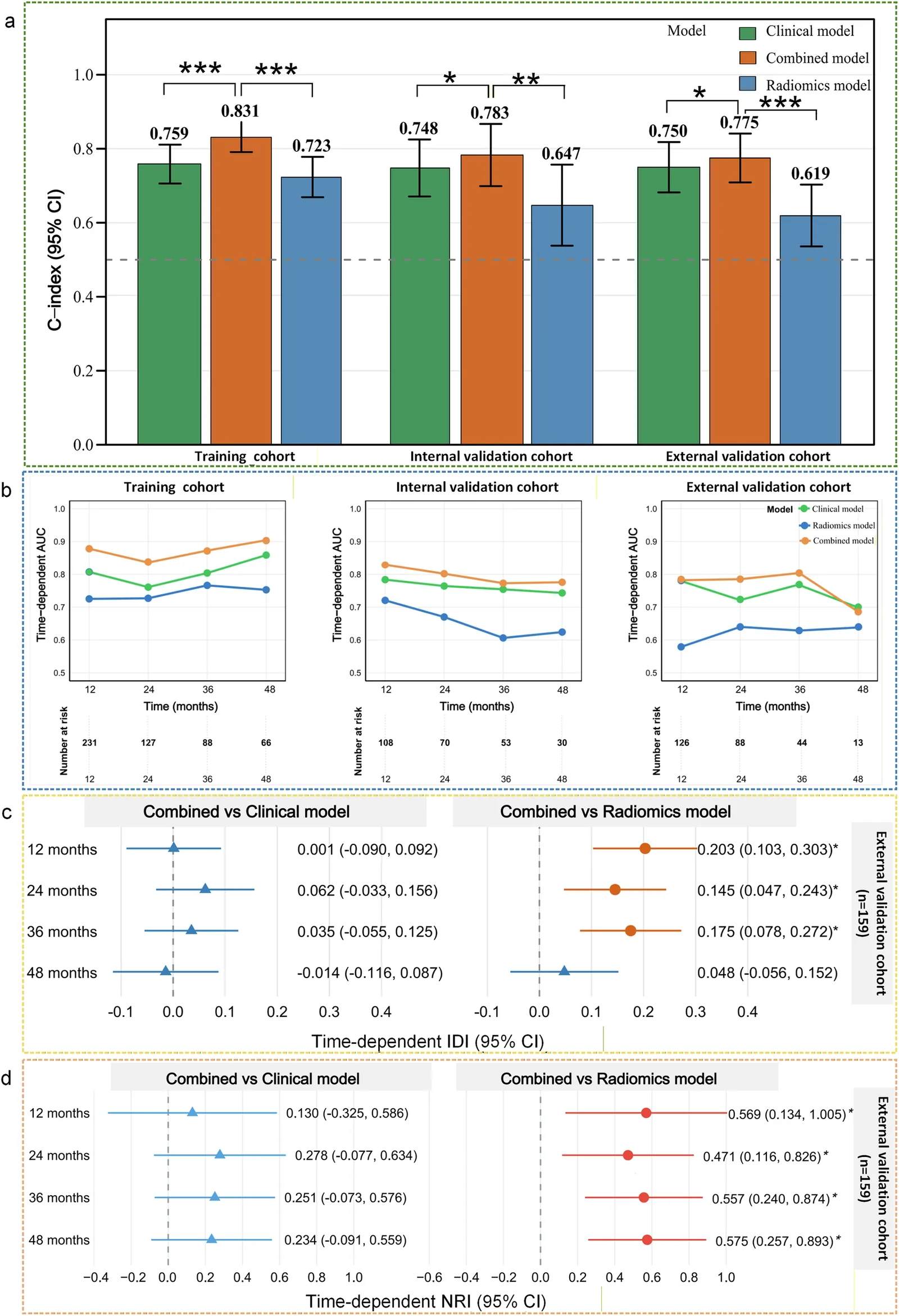
Model performance across the three datasets. **a** Harrell’s *C*-index for the clinical, radiomics, and combined models in the training, internal validation, and external validation cohorts. Error bars represent 95% confidence intervals (CIs). Asterisks indicate statistical significance (^*^p < 0.05, ^**^p < 0.01, ^***^p < 0.001). **b** Time-de*p*endent AUC at 12, 24, 36, and 48 months for the three models across the three datasets. The number of patients at risk at each time point is shown below the *x*-axis. **c** Time-dependent IDI for the combined model vs the clinical model (left) and the radiomics model (right) in the external validation cohort. Error bars represent 95% CIs. Solid orange circles and asterisks indicate statistically significant improvement (i.e., 95% CI excluding zero), while solid blue triangles indicate non-significant differences. **d** Time-dependent NRI for the combined model vs the clinical model (left) and the radiomics model (right) in the external validation cohort. Symbols are as in **c**

The combined model demonstrated moderate-to-good discriminative performance, with C-indices (95% CI) of 0.831 (0.791–0.872) in the training cohort, 0.783 (0.699–0.867) in the internal validation cohort, and 0.775 (0.709–0.841) in the external validation cohort (Fig. 3a and Table 2). The combined model consistently achieved higher C-indices across cohorts (Fig. S3).

**Table 2:** Discrimination performance of the predictive models

Model	*C*-index (95% CI)		
	Training cohort	Internal validation cohort	External validation cohort
Clinical model	0.759 (0.706, 0.811)	0.748 (0.671, 0.825)	0.750 (0.682, 0.818)
Radiomics model	0.723 (0.669, 0.778)	0.647 (0.538, 0.757)	0.619 (0.536, 0.703)
Combined model	0.831 (0.791, 0.872)	0.783 (0.699, 0.867)	0.775 (0.709, 0.841)

*CI* confidence interval

Model comparison demonstrated that the combined model had the highest log-likelihood (−356.91) and the lowest AIC (727.8) among the three models (Table S7). Furthermore, the multiphasic radiomics approach demonstrated superior discriminative performance compared to any single-phase radiomics model across all cohorts, confirming the incremental value of multiphasic integration (Table S8).

Time-dependent AUC analysis in the external validation cohort showed that AUCs for the combined model were maintained through 36 months but declined at 48 months, when the clinical model showed slightly higher AUC values (Fig. 3b and Table 3). Detailed AUC values are provided in Table S9 and Fig. S4.

**Table 3:** Time-dependent AUC of prediction models at different follow-up time points

Follow-up (months)	Model	AUC (95% CI)		
		Training cohort	Internal validation cohort	External validation cohort
12	Clinical model	0.807 (0.733, 0.882)	0.784 (0.660, 0.908)	0.781 (0.671, 0.891)
	Radiomics model	0.725 (0.644, 0.807)	0.721 (0.528, 0.914)	0.579 (0.454, 0.704)
	Combined model	0.877 (0.828, 0.925)	0.829 (0.691, 0.968)	0.782 (0.677, 0.888)
24	Clinical model	0.761 (0.687, 0.835)	0.764 (0.652, 0.877)	0.724 (0.627, 0.820)
	Radiomics model	0.727 (0.652, 0.802)	0.670 (0.521, 0.819)	0.640 (0.530, 0.750)
	Combined model	0.835 (0.775, 0.894)	0.802 (0.696, 0.908)	0.785 (0.699, 0.872)
36	Clinical model	0.804 (0.734, 0.874)	0.754 (0.638, 0.871)	0.769 (0.662, 0.876)
	Radiomics model	0.766 (0.693, 0.840)	0.606 (0.462, 0.751)	0.629 (0.507, 0.750)
	Combined model	0.871 (0.815, 0.927)	0.773 (0.660, 0.886)	0.804 (0.709, 0.899)
48	Clinical model	0.859 (0.799, 0.918)	0.743 (0.617, 0.870)	0.700 (0.524, 0.876)
	Radiomics model	0.753 (0.672, 0.834)	0.624 (0.482, 0.766)	0.638 (0.467, 0.809)
	Combined model	0.903 (0.852, 0.954)	0.776 (0.655, 0.897)	0.686 (0.502, 0.870)

*AUC* area under the curve, *CI* confidence interval

### Incremental predictive value

In the external validation cohort, the combined model showed higher predictive performance than the radiomics-only model across 12–36 months (e.g., 12-month IDI = 0.203 [95% CI: 0.103–0.303]; NRI = 0.569 [95% CI: 0.134–1.005]). Improvements over the clinical model were consistently positive but not statistically significant, indicating limited incremental gain from radiomics alone. The magnitude of improvement decreased over time (Fig. 3c, d). Complete comparisons of IDI and NRI across the three cohorts are presented in Fig. S5.

### Risk stratification and survival analysis

**Fig. 4:**
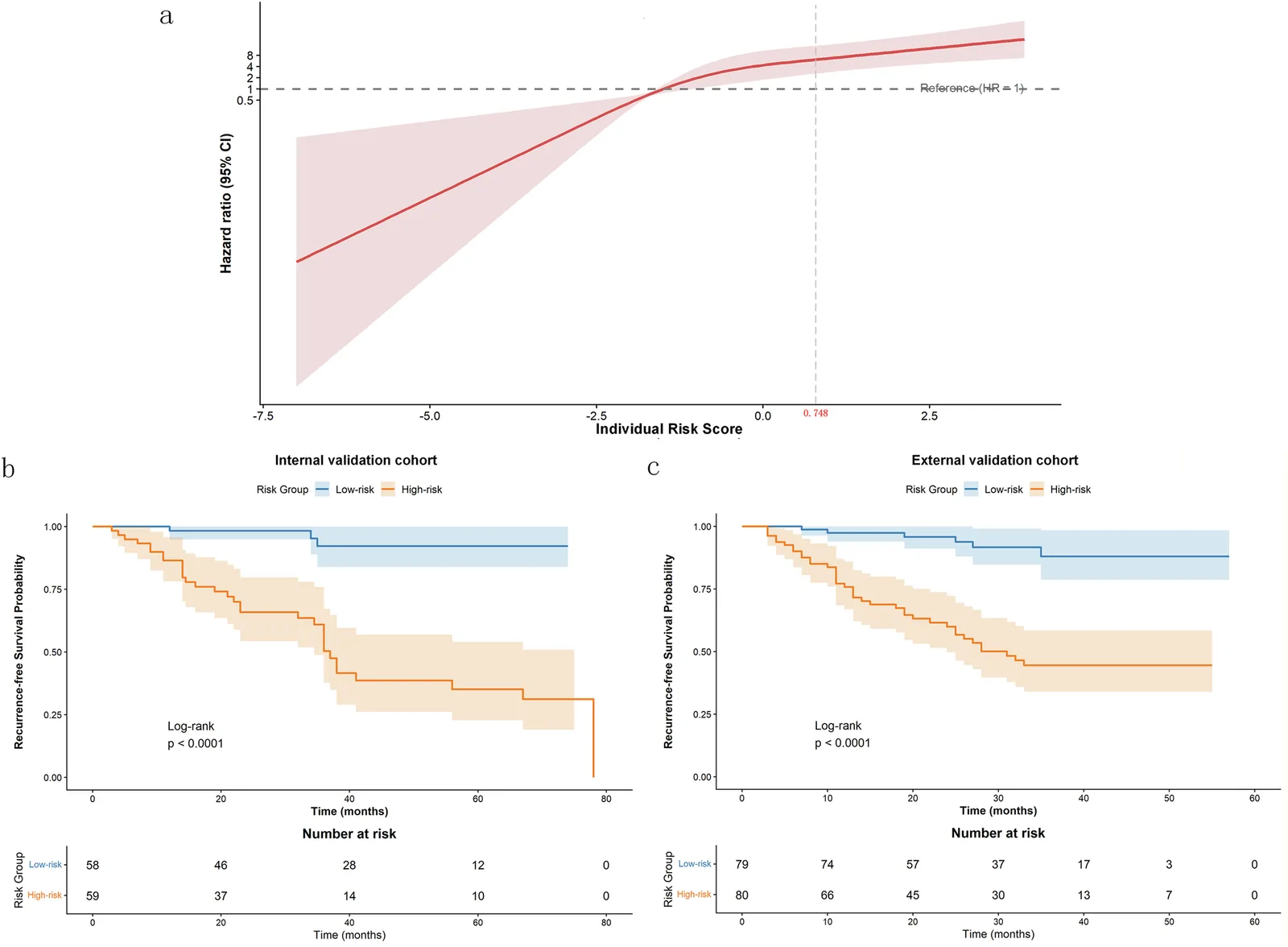
Risk stratification performance of the combined model. **a** RCS analysis in the training cohort showing the association between the individual risk score and the hazard ratio (HR) for recurrence. The solid line represents the fitted curve, with the shaded area indicating the 95% confidence interval. The horizontal dashed line denotes the reference hazard ratio (HR = 1), while the vertical dashed line marks the median individual risk score cutoff used for subsequent risk stratification. **b** Kaplan–Meier curves for recurrence-free survival in the internal validation cohort, stratified into high- and low-risk groups according to the median individual risk score cutoff from the training cohort. Numbers at risk are shown below the *x*-axis. Survival differences were compared using the log-rank test. **c** Kaplan–Meier curves for recurrence-free survival in the external validation cohort, using the same individual risk score formula and cutoff as in the training cohort. Numbers at risk are shown below the *x*-axis. Survival differences were compared using the log-rank test

RCS analysis demonstrated a continuous positive association between the combined model risk score and recurrence risk (Fig. 4a). Using the median training-cohort score as the cutoff, patients were stratified into high- and low-risk groups. The high-risk group had significantly lower recurrence-free survival than the low-risk group (log-rank *p* < 0.001; Fig. 4b, c), with median recurrence-free survival of 30.0, 36.0, and 27.0 months in the training, internal, and external validation cohorts, respectively; it was not reached in the low-risk group. The 3-year recurrence-free survival rate ranged from 86.8–92.7% in the low-risk group vs 37.4–49.7% in the high-risk group (Table 4). Similar trends were observed across the single-modality models (Fig. S6).

**Table 4:** Patient characteristics and survival outcomes stratified by the combined model

Cohort	Risk group	No. of patients	No. of events	Median RFS (months, 95% CI)	3-year RFS rate (%, 95% CI)	Log-rank *p*
Training cohort	Low-risk	136 (50.0%)	8 (5.9%)	NR	92.7 (87.7–98.0)	< 0.001
	High-risk	136 (50.0%)	74 (54.4%)	30.0 (21.0–42.0)	43.6 (34.9–54.4)	
Internal validation cohort	Low-risk	62 (53.0%)	5 (8.1%)	NR	89.6 (81.1–99.1)	< 0.001
	High-risk	55 (47.0%)	30 (54.5%)	36.0 (34.5–56.0)^a^	49.7 (36.6–67.7)	
External validation cohort	Low-risk	93 (58.5%)	9 (9.7%)	NR	86.8 (78.6–96.0)	< 0.001
	High-risk	66 (41.5%)	36 (54.5%)	27.0 (20.0–33.0)^a^	37.4 (26.3–53.1)	

*RFS* recurrence-free survival, *CI* confidence interval, *NR* not reached

^a^ 95% confidence intervals were estimated using the bootstrap method with 1000 resamples

### Calibration and clinical utility

**Fig. 5:**
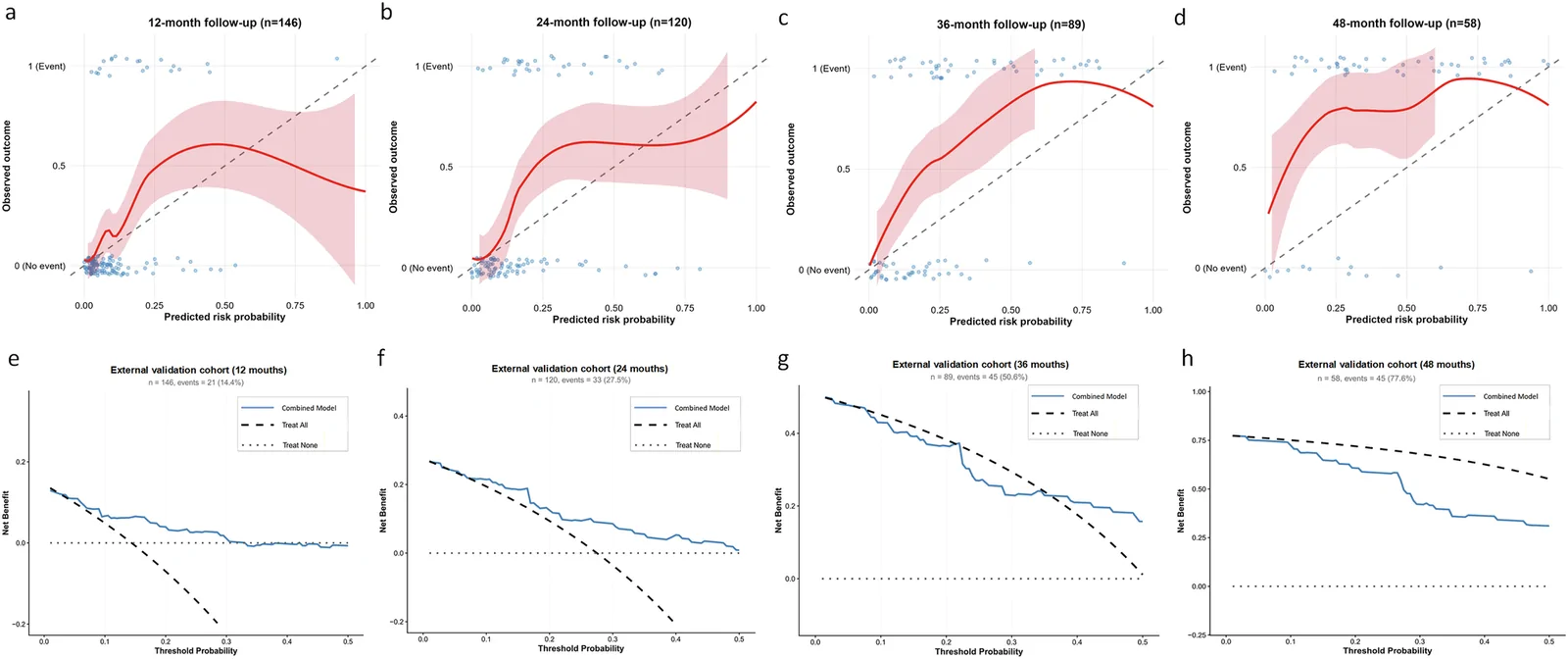
Calibration and clinical utility of the combined model in the external validation cohort. **a**–**d** Patient-level calibration curves at 12, 24, 36, and 48 months, respectively. Each dot represents an individual patient. The red solid line indicates the smoothed calibration curve, with the shaded area representing the 95% confidence interval (CI). The black dashed diagonal line represents perfect calibration. **e**–**h** DCA at 12, 24, 36, and 48 months, respectively. The *x*-axis denotes the threshold probability, and the *y*-axis denotes the net benefit. The net benefit of three strategies is shown: the combined model (solid blue line), the treat all strategy (gray dashed line), and the treat none strategy (gray dotted line)

The combined model showed imperfect calibration at 12 months (calibration slope = 0.506; Brier score = 0.114; Hosmer–Lemeshow, *p* = 0.072). Calibration performance varied over time, with slopes of 0.730 at 24 months and 0.936 at 36 months, followed by a decline to 0.516 at 48 months, when the Hosmer–Lemeshow test remained significant (*p* < 0.001). The Brier score and ICI increased from 0.114 and 0.188 at 12 months to 0.308 and 0.476 at 48 months, respectively. The number of patients at risk decreased from 146 to 58 over the same period (Table 5 and Fig. 5a–d).

**Table 5:** Calibration and clinical utility of the combined model at different time points in the external validation cohort

Time point (months)	No. of patients	Calibration performance				Clinical utility (threshold = 0.2)^a^	
		Calibration slope	Brier score	ICI	HL *p*-value	Net benefit	Comparison with ‘Treat All’
12	146	0.506	0.114	0.188	0.072	0.0394	Higher
24	120	0.73	0.176	0.288	**0.003**	0.125	Higher
36	89	0.936	0.234	0.374	**< 0.001**	0.3652	Slightly lower
48	58	0.516	0.308	0.476	**< 0.001**	0.6078	Lower

*ICI* integrated calibration index, *HL* Hosmer–Lemeshow test

Values in bold indicate statistical significance (*p* < 0.05)

^a^ A threshold probability of 0.2 was used as the clinically relevant decision threshold for DCA. Net benefit values are presented as absolute numbers

DCA demonstrated that the combined model provided net benefit over treat-all and treat-none strategies at 12 and 24 months. At longer time horizons, the net benefit decreased, and the treat-all strategy showed greater net benefit at higher thresholds (Table 5 and Fig. 5a–h).

### Model interpretation and case illustrations

**Fig. 6:**
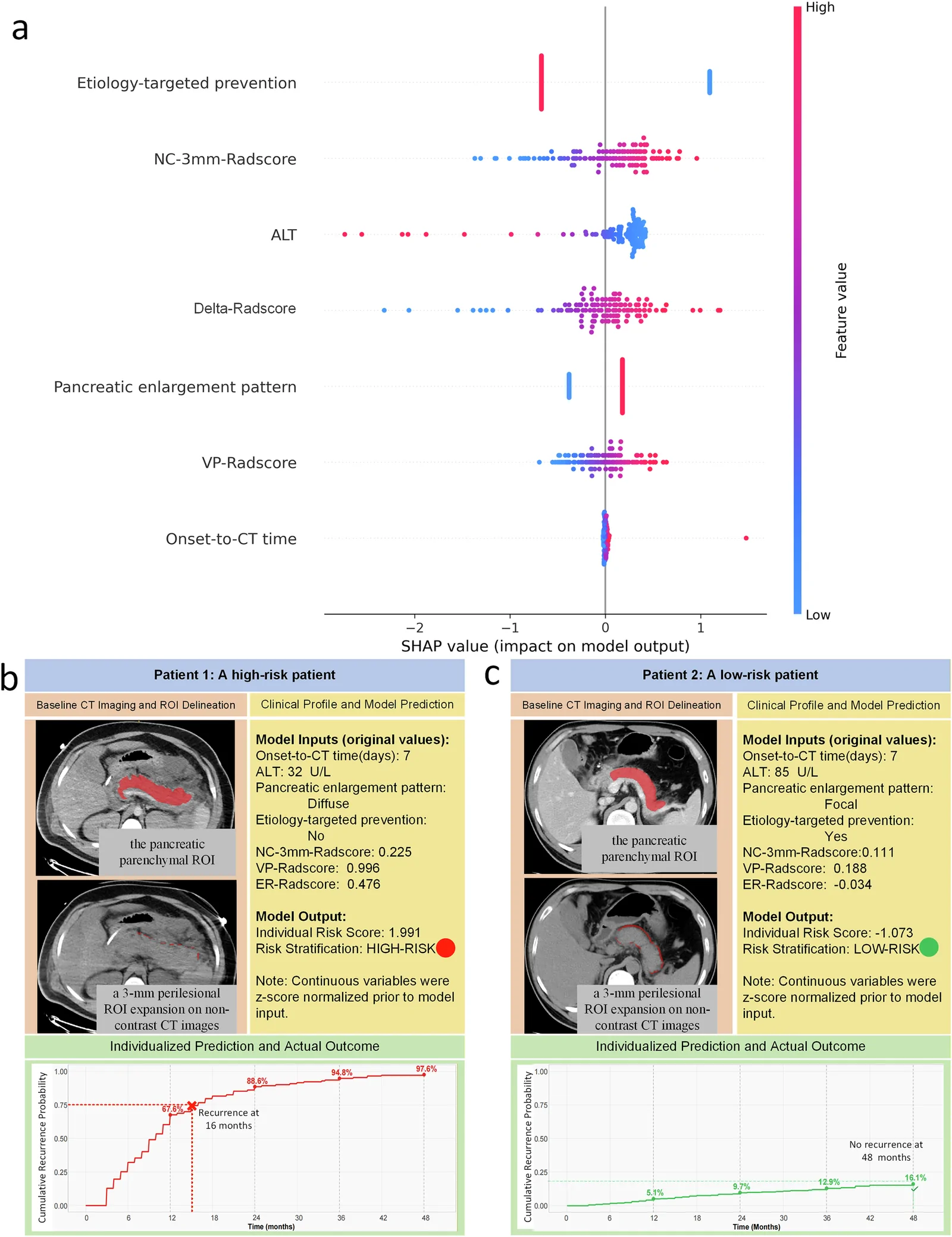
Model interpretability and individualized risk prediction. **a** SHAP summary plot showing the contributions of the top seven predictors in the combined model. Features are ranked by mean absolute SHAP value (global importance). Positive SHAP values: higher risk; negative: lower risk. Each point represents a patient from the training cohort, with color indicating feature value (red: high, blue: low). For categorical variables, red indicates “no etiology-targeted prevention” and “diffuse pancreatic enlargement”, while blue indicates “yes” and “focal/no enlargement”. **b** High-risk patient from the external validation cohort with recurrence at 16 months. Baseline CT image shows the segmented pancreatic parenchymal ROI. The solid red line represents the individualized cumulative recurrence probability curve, with the red dashed vertical line marking the recurrence event. **c** Low‑risk patient from the external validation cohort with no recurrence during 48 months of follow‑up. Baseline CT image and cumulative recurrence probability curve (solid green line) are shown. The green dashed vertical line indicates the censoring time. Continuous variables were *z*-score normalized prior to model input. NC-3mm-Radscore, radiomics score of the peripancreatic region extending 3 mm outward on NC CT; Delta-Radscore, radiomics score derived from the percentage change between contrast-enhanced (arterial and portal venous phase) and NC radiomics features; VP-Radscore, portal venous phase radiomics score; ALT, alanine aminotransferase; SHAP, Shapley additive explanations

SHAP analysis identified etiology-targeted prevention contributed most to risk reduction, whereas NC-3mm-Radscore contributed most to risk increase, consistent with its hazard ratio (HR = 3.123). Higher ALT levels were associated with lower recurrence risk. Other contributors included Delta-Radscore, pancreatic enlargement pattern, VP-Radscore, and onset-to-CT time (Figs. 6a and S7).

Representative cases further illustrated model performance. A high-risk patient (risk score = 1.991) developed recurrence at 16 months, whereas a low-risk patient (risk score = −1.073) remained recurrence-free at 48 months, consistent with model predictions (Figs. 6b, c and S8).

## Discussion

In this multicenter study, we developed and externally validated a combined model integrating multiphase CT radiomics with key clinical variables to predict recurrence risk after first-episode AP. Unlike prior studies relying on single-phase or single-modality imaging approaches [12, 15, 16], our approach leverages multiphase contrast-enhanced CT to capture complementary structural and enhancement-related information. As demonstrated in our comparative analysis (Table S8), and consistent with recent evidence utilizing dynamic Delta radiomics [27–29], the multiphasic integration provided improved predictive performance over single-phase models, enabling a more comprehensive characterization of disease heterogeneity. Among radiomics features, the NC-3mm-Radscore and Delta-Radscore emerged as the strongest predictors of recurrence, capturing complementary structural and enhancement-related characteristics. The NC-3mm-Radscore was associated with recurrence risk and may capture subtle structural alterations in the peripancreatic region, whereas the Delta-Radscore incorporated dynamic enhancement information associated with temporal contrast variation. Importantly, the model enables individualized recurrence probability estimation, facilitating patient-specific risk stratification.

Recurrence after acute pancreatitis may be driven, in part, by structural microenvironmental alterations. Radiomics may enable the detection of subtle alterations in peripancreatic fat beyond conventional visual assessment [10]. The findings suggest that the 3-mm peripancreatic region may contain imaging information associated with recurrence susceptibility, potentially reflecting localized alterations in the peripancreatic tissue environment. This finding complements the established role of intra-pancreatic fat deposition (IPFD) as an important factor associated with disease onset [30, 31], whereas peripancreatic fat may represent a tissue component associated with local inflammatory changes. Together, these findings suggest that abnormalities in both compartments may contribute to recurrence risk. Incorporating Delta-Radscore provides additional imaging interpretability through dynamic contrast-enhancement patterns. Unlike static features, Delta-Radscore provides quantitative information regarding temporal enhancement changes, which may reflect alterations in tissue perfusion characteristics [32]. Further studies integrating radiological, pathological, and molecular data are required to clarify the biological basis underlying these radiomic signatures. The independent prognostic value of Delta-Radscore suggests that dynamic enhancement features may provide additional imaging information associated with recurrence risk.

Among clinical variables, etiology-targeted prevention emerged as a protective factor associated with reduced recurrence risk [2, 17]. The protective effect of ALT may be interpreted through two complementary mechanisms: resolution of biliary etiology [7] and alterations in metabolic status [2]. These findings suggest that hepatic function and metabolic status may be associated with recurrence risk, although the underlying mechanisms require further investigation.

Compared with prior studies relying on single-modality imaging or clinical variables alone [12, 15, 16, 33], this study integrates multiphase CT radiomics with clinical data for recurrence risk stratification, quantifies peripancreatic features, and incorporates Delta-Radscore to capture dynamic imaging characteristics. Model development followed TRIPOD guidelines [24]. The multicenter design enhances model generalizability [34]. Model performance was comprehensively evaluated using multiple complementary metrics, and SHAP analysis enhanced interpretability, supporting clinical transparency in multicenter radiomics studies [25, 35]. In addition, inclusion of onset-to-CT time helped mitigate temporal bias [23]. Time-dependent IDI/NRI analysis suggested that the incremental value of the combined model was most evident within the first 36 months, coinciding with the peak recurrence period. Improvement over the radiomics model was significant (12-month IDI = 0.203). While improvement over the clinical model was not statistically significant, the positive point estimates suggest potential incremental value of radiomics, although the magnitude of improvement should be interpreted cautiously. It is noteworthy that the radiomics-only model showed suboptimal performance in the external validation cohort (AUC = 0.579 at 12 months). This likely reflects the fact that AP recurrence is a process heavily driven by post-discharge clinical interventions (e.g., etiology-targeted prevention such as lipid-lowering therapy or alcohol cessation). While radiomics may provide imaging-derived information related to localized tissue characteristics associated with recurrence risk, it cannot account for subsequent dynamic etiological interventions. This highlights the importance of integrating multiphasic radiomics with etiology-targeted prevention when developing recurrence prediction models.

In the external validation cohort, 3-year recurrence-free survival was 37.4% in the high-risk group vs 86.8% in the low-risk group. This clear separation guides clinical management: high-risk patients may benefit from intensified surveillance (e.g., targeted etiological interventions and closer imaging follow-up), while low-risk patients can be managed with routine follow-up, optimizing resource allocation [36]. Given that recurrent AP reduces quality of life [2], accurate risk identification is clinically crucial. DCA indicated that the combined model provided superior net benefit over “treat-all” and “treat-none” strategies within the 12- to 24-month window, supporting early precision intervention. SHAP-based risk score derivation enhances transparency and facilitates individualized management [25, 26].

The combined model showed acceptable calibration at 12 months, but calibration performance declined with longer time horizons, likely due to decreasing sample size (146 at 12 months vs 58 at 48 months), competing risks (e.g., progression to chronic pancreatitis), and the inherent limitations of static models [23]. Despite suboptimal long-term calibration, the model retained potential clinical utility, supported by strong discrimination and favorable DCA profiles. The low-risk group maintained > 85% 3-year recurrence-free survival, suggesting reliable identification of patients who do not require intensive surveillance. Future improvements may involve probability recalibration or dynamic modeling approaches.

This study has several limitations. First, its retrospective design may introduce selection bias. Second, manual correction steps in image segmentation may lead to interobserver variability. Third, as a static model, its long-term predictive performance may be limited. Finally, the confidence intervals for IDI/NRI were relatively wide. These limitations underscore the need for further investigation through prospective validation, standardized automated workflows, dynamic modeling approaches, and large-scale multicenter collaborations [23, 34, 35].

In conclusion, this multicenter study developed and externally validated a combined model integrating multiphase CT radiomics with clinical variables to predict recurrence risk after first-episode AP. The 3-mm peripancreatic region was identified as an important imaging feature associated with recurrence risk, while Delta-Radscore provided additional prognostic information through dynamic contrast-enhancement features. By generating individualized recurrence probability curves, the model supports risk stratification and secondary prevention strategies.

## Supplementary information

Electronic supplementary material

## Data Availability

The datasets generated and/or analyzed during the current study are not publicly available due to institutional privacy restrictions but are available from the corresponding author on reasonable request.

## Abbreviations

AIC: Akaike information criterion
ALT: Alanine aminotransferase
AP: Acute pancreatitis
AUC: Area under the curve
CECT: Contrast-enhanced computed tomography
*C*-index: Concordance index
DCA: Decision curve analysis
IDI: Integrated discrimination improvement
LASSO: Least absolute shrinkage and selection operator
NC: Non-contrast
NRI: Net reclassification improvement
RAP: Recurrent acute pancreatitis
RCS: Restricted cubic spline
ROI: Region of interest
SHAP: Shapley additive explanations
VP: Venous phase
