# Supplementary material for: Multiphasic CT radiomics–clinical model for predicting acute pancreatitis recurrence: a multicenter survival analysis
Source: Insights Imaging. 2026 Nov 4;17:280. doi: 10.1186/s13244-026-02439-6 (PMC13737284; doi:10.1186/s13244-026-02439-6)
Supplement: Supplementary file 1 [file 13244_2026_2439_MOESM1_ESM.pdf]

Multiphasic CT radiomics–clinical model for predicting acute pancreatitis recurrence: a multicenter survival analysis

ELECTRONIC SUPPLEMENTARY MATERIAL

Contents

Supplementary Method

Supplementary Method 1. CT image feature assessment criteria

Supplementary Method 2. Detailed CT image preprocessing

Supplementary Method 3. Development and evaluation of an automated pancreas segmentation model using nnU-Net

Supplementary Method 4. Radiomic feature extraction

Supplementary Method 5. Delta-Radscore

Supplementary Method 6. Control of temporal bias

Supplementary Method 7. Calculation of IDI and NRI

Supplementary Method 8. Generation of individualized recurrence probability curves

Supplementary Result

Supplementary Result 1. Restricted cubic spline analysis of continuous variables

Supplementary Result 2. Sensitivity analysis of model variable selection

Supplementary Table

Table S1. Performance of the nnU-Net-based pancreas segmentation model

Table S2. Baseline characteristics of the study cohorts

Table S3. Multivariable Cox regression analysis of the clinical model in the training cohort

Table S4. Multivariable Cox regression analysis of the radiomics model in the training cohort

Table S5. Detailed characteristics and standardized coefficients of selected radiomics features for constructing the NC-3mm, VP, and Delta Radscores

Table S6. Impact of the pancreatic enlargement pattern on model discriminatory performance

Table S7. Comparison of model fit statistics in the training cohort

Table S8. Comparison of predictive performance between single-phase and multiphasic radiomics models across study cohorts (adjusted for onset-to-CT time)

Table S9. Time-dependent AUC of prediction models at different follow-up time points

Supplementary Figure

Figure S1. Restricted cubic spline (RCS) analyses of individual continuous predictors in the training cohort

Figure S2. Baseline recurrence-free survival across the three study cohorts

Figure S3 Forest plot of the C-index for each prediction model across the three study cohorts

Figure S4. Time-dependent AUC for each prediction model across the three study cohorts

Figure S5. Incremental value of the combined model

Figure S6. Direct comparison of risk stratification by the three models in the validation cohorts

Figure S7. Forest plot of multivariable Cox regression analysis in training cohort

Figure S8. SHAP waterfall plots and force plots illustrating the feature contribution to Patient 1 (high-risk) and Patient 2 (low-risk)

Supplementary Method

Supplementary Method 1. CT image feature assessment criteria

All CT images were independently evaluated by two abdominal radiologists with over 10 years of experience, both blinded to clinical and follow-up data. A standardized training session was conducted prior to assessment to ensure consistency. Any disagreements were resolved by consensus. The detailed assessment criteria are as follows:

(1) Morphological type of acute pancreatitis (AP)

Interstitial edematous pancreatitis: Inflammatory changes in the pancreatic parenchyma or peripancreatic fat without evidence of parenchymal necrosis.

Necrotizing pancreatitis: Presence of non-enhancing necrotic areas within the pancreatic parenchyma and/or peripancreatic tissues.

(2) Type of pancreatic enlargement

Based on morphological changes, pancreatic enlargement was categorized as:

Focal enlargement: Localized enlargement with normal appearance of the remaining parenchyma.

Diffuse enlargement: Homogeneous or heterogeneous enlargement involving the entire pancreas.

(3) Pleural effusion

Defined as the presence of fluid density in the unilateral or bilateral thoracic cavity (present/absent).

(4) Peritoneal effusion

Defined as the presence of fluid density within the peritoneal cavity (e.g., hepatorenal recess, splenorenal recess, mesenteric spaces) (present/absent).

(5) Modified CT severity index (mCTSI)

The mCTSI was scored based on pancreatic and peripancreatic changes, extent of pancreatic necrosis, and extra-pancreatic complications, with total scores ranging from 0 to 10. The detailed scoring criteria are as follows:

Pancreatic inflammation/fluid collections:

- – Normal pancreas (0 points)
- – Focal or diffuse enlargement of the pancreas, with or without peripancreatic fat stranding (2 points)
- – Single or multiple fluid collections within the pancreas or peripancreatic tissues, or peripancreatic fat necrosis (4 points)

Pancreatic necrosis:

- – No pancreatic necrosis (0 points)
- – Necrosis extent ≤30% (2 points)
- – Necrosis extent >30% (4 points)

Extra-pancreatic complications: Includes pleural effusion, peritoneal effusion, vascular

complications, gastrointestinal involvement, etc. The presence of any one complication adds 2 points (maximum 2 points).

The above criteria were based on the 2012 Atlanta classification and relevant literature [1–3].

Supplementary Method 2. Detailed CT image preprocessing

To reduce scanner- and protocol-related variability—factors known to affect radiomic feature reproducibility [4]—a standardized preprocessing workflow was applied to all non-contrast, arterial phase, and venous phase images from both institutions. Preprocessing was performed using the Shukun Research Platform (Shukun Technology, Beijing, China; https://medresearch.shukun.net), whose routines are built upon the PyRadiomics framework. Raw images were initially acquired with a slice thickness of 1–1.25 mm and subsequently reconstructed to 5 mm for clinical evaluation and downstream analysis. The reconstructed 5-mm images had variable in-plane resolutions.

The preprocessing sequence comprised the following steps:

Image loading and orientation. Original patient alignment encoded in the DICOM headers was preserved.

Resampling. All reconstructed 5-mm volumes were resampled to an isotropic voxel size of 1 × 1 × 1 mm^3 using linear interpolation; voxels falling outside the original field of view were assigned a value of −1024 HU.

Gray-level discretization. To harmonize intensity distributions and reduce noise, gray levels were quantized using a fixed bin width of 25 HU within the range of –1000 HU to +1000 HU; values outside this window were truncated to the nearest bound prior to binning.

Intensity normalization. Per-image z-score normalization was performed using the mean and standard deviation derived exclusively from voxels within the abdominal cavity (air excluded), yielding images with zero mean and unit variance.

Every preprocessed dataset underwent visual inspection to confirm proper alignment and absence of processing-induced artifacts. These standardized images served as the basis for subsequent pancreatic parenchyma segmentation (Supplementary Method 3) and extraction of regions of interest, which included the whole pancreatic parenchyma on NC, AP, and PVP series, as well as 3-mm and 5-mm peripancreatic expansion zones on NC images.

Supplementary Method 3. Development and evaluation of an automated pancreas segmentation model using nnU-Net

Pancreatic parenchyma segmentation was performed using a deep learning model based on the nnU-Net framework (version 2) [5]. As a self-configuring segmentation framework, nnU-Net automatically adapts preprocessing, network architecture, training strategy, and post-processing to the characteristics of the input data, enabling robust and standardized pancreas segmentation. The source code is publicly available at https://github.com/MIC-DKFZ/nnUNet.

Image preprocessing prior to segmentation

Following the default configuration of nnU-Net v2, a dedicated preprocessing pipeline was applied to all 5-mm reconstructed CT images (the same 5-mm images used for clinical assessment and ROI definition) to optimize them for pancreas segmentation.
Resampling: Images were resampled to a voxel spacing of $5.0 \times 0.8 \times 0.8$ mm^3, automatically determined by the nnU-Net framework for this dataset.
Intensity clipping: Intensity values were clipped between the 0.5th and 99.5th percentiles to minimize the impact of extreme outliers.
Z-score normalization: Intensity values were normalized to zero mean and unit variance to improve training stability and cross-dataset generalizability.
Model training setup
The segmentation model employed a 3D full-resolution U-Net architecture using the default nnU-Net v2 configuration.Training was guided by a composite loss function combining Dice loss and cross-entropy loss. A total of 1000 training epochs were executed with a batch size of 2. Optimization was performed using stochastic gradient descent with an initial learning rate of 0.01 and Nesterov momentum of 0.99. To improve generalization and robustness, extensive data augmentation—including random rotation, scaling, cropping, and flipping—was applied during training. All model training and inference were implemented in the PyTorch framework using an NVIDIA RTX 4090D GPU.
Dataset partitioning and performance metrics
A total of 60 cases were used for model development. A five-fold cross-validation strategy was adopted for internal validation. To further evaluate generalizability, an independent external test cohort comprising 26 cases from center B was additionally included. Segmentation performance was assessed using the Dice Similarity Coefficient (DSC), Intersection over Union (IoU), Positive Predictive Value (PPV), and Sensitivity. The performance results for both the internal cross-validation and the external test cohort are summarized in Supplementary Table 1.

Supplementary Method 4. Radiomic feature extraction
Radiomic feature extraction was performed using the PyRadiomics engine, as implemented on the Shukun Research Platform (Shukun Technology, Beijing, China; https://medresearch.shukun.net), following the reference standard described in its official documentation (van Griethuysen et al., 2017; https://pyradiomics.readthedocs.io/). For each patient, features were extracted from three distinct regions of interest (ROIs): the entire pancreatic parenchyma on non-contrast (NC), arterial phase (AP), and portal venous phase (PVP) images, as well as from 3-mm and 5-mm peripancreatic expansion regions on NC images.
A total of 1,874 radiomic features were extracted per ROI per phase. This number comprises features calculated from the original image and a series of derived images. From the original image, 107 features were extracted across seven families: 18 first-order statistics, 24 gray-level co-occurrence matrix (GLCM) features, 14 gray-level dependence matrix (GLDM) features, 16 gray-level run-length matrix (GLRLM) features, 16 gray-level size zone matrix (GLSZM) features, 5 neighborhood gray-tone difference matrix (NGTDM) features, and 14 shape features (calculated only from the original image). To

enrich the feature set, the same 93 first-order and texture features (all except shape) were also extracted from 19 additional derived images, including 8 wavelet sub-bands, 2 Laplacian of Gaussian (LoG) filtered images (with two sigma values), and one each of exponential, logarithm, square, square root, gradient, local binary pattern 2D (LBP2D), and three modes of local binary pattern 3D (LBP3D). Detailed definitions of these filters and derived image types are available in the PyRadiomics documentation (https://pyradiomics.readthedocs.io/en/latest/). The total feature count per ROI was therefore calculated as (18 + 24 + 14 + 16 + 16 + 5) × 20 + 14 = 1,874, where 20 represents the total number of image types (original plus 19 derived images).

Supplementary Method 5. Delta-Radscore

To quantitatively capture the dynamic changes of radiomic features during contrast enhancement, enhancement-related features were derived by calculating the relative change between the non-contrast (NC) and contrast-enhanced phases [6-8]. For any radiomic feature f, its relative change Δf_{phase} for a given phase was defined as:

$$\Delta f_{phase}=\frac{f_{phase}-f_{NC}}{f_{NC}}\times 100\%,\quad phase\in\{AP,VP\}$$

where f_{NC}, f_{AP}, and f_{VP} denote the original feature values extracted from non-contrast, arterial phase (AP), and portal venous phase (VP) images, respectively. Using the raw feature values, a total of 3,748 enhancement-derived features (1,874 features × 2 phases) were generated. To account for different feature scales and ensure fair penalization during the subsequent LASSO regression, all derived Delta features were then z-score standardized (mean = 0, standard deviation = 1) across the training cohort prior to feature screening.

Feature Cleaning and Initial Screening

The initial feature set was cleaned by removing redundant features—those with identical values despite different names—ensuring that each retained feature carried independent information. Features were then screened for redundancy by calculating pairwise Pearson correlation coefficients. For any pair of features with an absolute correlation $|r|>0.9$, the feature with the smaller *p*-value in univariate Cox regression analysis for recurrence-free survival was retained, and the other was excluded.

Stability Selection via Bootstrap Resampling and Lasso-Cox

To identify features with stable associations with recurrence prognosis, a stability selection procedure was applied. One hundred bootstrap samples were drawn with replacement from the training cohort, each with the same size as the original training cohort. For each sample, a Lasso-Cox regression model was fitted, with the optimal penalization parameter λ\\lambdaλ determined by 10-fold cross-validation. The selection frequency of each feature (i.e., the proportion of bootstrap samples in which its coefficient was non-zero) was recorded.

Based on the number of recurrence events in the training cohort (n = 82) and the “10–20 events per variable” rule, the expected number of predictors in the final model was approximately eight. Features were ranked by selection frequency, and a cutoff was determined by inspecting the frequency distribution for an inflection point. The top 8 to 12

features with the highest frequencies were retained as “stable features.” The exact number of stable features retained is reported in the Results section.

Construction of Delta-Radscore

From the set of stable features, a multivariable Cox regression model with backward elimination was applied to identify features independently associated with recurrence risk. Features with a retention criterion of $p < 0.05$ were retained in the final subset. A multivariable Cox model without penalization was then fitted to the entire training cohort using this feature subset. The resulting regression coefficients β_i were used to construct the Delta-Radscore as a weighted linear combination:

$$\text{Delta−Radscore} = \sum_{i=1}^{k} \beta_i \cdot \Delta f_i$$

where Δf_i represents the relative change value of the i-th stable feature (derived from either the arterial or portal venous phase), and k is the total number of features in the final subset. This score integrates dynamic enhancement information from both contrast-enhanced phases and captures the overall pattern of radiomic feature changes during contrast enhancement.

Comparison of Construction Strategies

To validate the stability selection approach, principal component analysis (PCA) was performed on the same set of enhancement-related features after initial screening. The first few principal components accounting for > 70% of the cumulative variance were extracted as alternative predictors. The prognostic discrimination of these PCA-derived components and the stability-selected features was compared in multivariable Cox models using the C-index. The difference in C-index between the two strategies was < 0.01 in the training cohort, indicating highly similar predictive performance. Considering model parsimony and consistency with the main radiomics analysis, the Delta-Radscore derived from stability selection was chosen as the representative enhancement-related feature for inclusion in subsequent survival models.

Supplementary Method 6. Control of temporal bias

To mitigate the potential confounding effect of the interval from symptom onset to CT examination (onset-to-CT time), this variable was prespecified as a mandatory adjustment covariate in all final Cox models (clinical, radiomics, and combined). It was included in the models without undergoing any feature selection process, thereby preventing data leakage and enabling an unbiased assessment of the independent contributions of other predictors [4].

The rationale for this methodological choice is twofold. First, the CT manifestations of acute pancreatitis (AP) are highly time-dependent [9]. Scans acquired early after onset (e.g., within 72 h) may not fully depict pancreatic necrosis or peripancreatic fluid collections, whereas delayed scans may capture partial resolution of inflammatory changes, both of which can affect the stability of radiomic features [10]. Second, the timing of CT examinations is inherently heterogeneous in clinical practice, influenced by factors such as hospital workflows, patient acuity, transportation logistics, and clinical judgment. As these factors may correlate with baseline patient characteristics (e.g.,

etiology, disease severity), failing to adjust for onset-to-CT time could introduce substantial confounding bias.

To ensure methodological consistency and fair comparison across models, onset-to-CT time was uniformly adjusted in all candidate models. This strategy aligns with international consensus in radiomics research; for instance, Avanzo et al. recommend that "for key confounding factors, especially time-dependent variables, it is advisable to prespecify and forcibly include them in multivariable analysis to ensure the independent prognostic value of radiomic features" [4]. By mandatorily adjusting for onset-to-CT time in all models, this study aims to accurately evaluate the independent contribution of each predictor to AP recurrence while minimizing potential biases arising from multi-center data collection and clinical practice heterogeneity.

Supplementary Method 7. Calculation of IDI and NRI

The incremental predictive value of the combined model over the clinical and radiomics models was quantified using the integrated discrimination improvement (IDI) and the continuous net reclassification improvement (NRI) [11].

IDI

The IDI assesses the overall improvement in predicted probabilities between a new and an old model. It is defined as the difference in the mean predicted probabilities for events and non-events between the two models:

$$IDI=[P_{new(event)}-P_{old(event)}]-[P_{new(non-event)}-P_{old(non-event)}]$$

Among them, P_{new} and P_{old} are the mean predicted probabilities from the new and old models for the event and non-event groups, respectively. A positive IDI indicates improved discrimination of the new model.

NRI

The continuous NRI evaluates the correctness of changes in predicted probabilities without requiring a predefined risk cutoff. It is calculated as:

$$NRI=[P(up|event)-P(down|event)]-[P(up|non-event)-P(down|non-event)]$$

where "up" denotes a higher predicted probability from the new model compared to the old, and "down" denotes a lower predicted probability. The NRI thus measures the net proportion of events correctly assigned a higher risk and non-events correctly assigned a lower risk by the new model.

Implementation and statistical inference

For each comparison (combined model vs. clinical model and combined model vs. radiomics model), IDI and NRI were calculated at four prespecified follow-up time points: 12, 24, 36, and 48 months. The 95% confidence intervals for both metrics were obtained using 1000 bootstrap resamples [11]. All analyses were performed using the nricens package in R (version 4.5.2). A two-sided $p < 0.05$ was considered statistically significant.

Supplementary Method 8. Generation of individualized recurrence probability curves

To visually demonstrate the individualized predictive ability of the combined Cox model, one high-risk and one low-risk patient were selected from the external validation cohort. For each, an individualized cumulative recurrence probability curve was generated as follows.

Individual risk score calculation. The raw values of the clinical and radiomic features for the selected patients were first z-score standardized using the mean and standard deviation from the training cohort. These standardized values were then input into the trained combined Cox model, and the individual risk score was obtained using the predict function in R.

Individualized survival probability estimation. The survfit function from the R survival package combined the model’s baseline survival function with the individual risk score to predict monthly survival probabilities over the 0- to 48-month follow-up period. Cumulative recurrence probability at each time point was defined as 1 minus the survival probability.

Probability curve generation. A smoothed curve was plotted for each patient, with follow-up time (months) on the x-axis and cumulative recurrence probability on the y-axis. Key time points (12, 24, 36, and 48 months) were marked on the curve and annotated with corresponding predicted probability values.

Overlay of actual outcomes. To provide a visual comparison between predicted and actual outcomes, the timing of the recurrence event (for the high-risk patient) is indicated by a red vertical dashed line; for the censored low-risk patient (no recurrence), the last follow-up time point is marked with a green checkmark.

All figures were generated using the R packages ggplot2 and cowplot.

Supplementary References

1. Banks PA, Bollen TL, Dervenis C, et al (2013) Classification of acute pancreatitis--2012: revision of the Atlanta classification and definitions by international consensus. Gut 62(1):102-111
2. Bollen TL, Singh VK, Maurer R, et al (2011) Comparative evaluation of the modified CT severity index and CT severity index in assessing severity of acute pancreatitis. AJR Am J Roentgenol 197(2):386-392
3. Tenner S, Vege SS, Sheth SG, et al (2024) American College of Gastroenterology Guidelines: Management of Acute Pancreatitis. Am J Gastroenterol 119(3):419-437
4. Avanzo M, Soda P, Bertolini M, et al (2026) Robust radiomics: a review of guidelines for radiomics in medical imaging. Front Radiol 5:1701110
5. Isensee F, Jaeger PF, Kohl SAA, Petersen J, Maier-Hein KH (2021) nnU-Net: a self-configuring method for deep learning-based biomedical image segmentation. Nat Methods 18(2):203-211
6. Wang W, Jin F, Song L, et al (2025) Prediction of peripheral lymph node metastasis (LNM) in thyroid cancer using delta radiomics derived from enhanced CT combined with multiple machine learning algorithms. Eur J Med Res 30(1):164

7. Li H, Zhang L, Song L, et al (2024) Delta-CT radiomics based model for predicting postoperative anastomotic leakage following radical resection of esophageal squamous cell carcinoma. Front Oncol 14:1485323
8. Wu X, Yang J, Ji X, et al (2025) Delta radiomics modeling based on CTP for predicting hemorrhagic transformation after intravenous thrombolysis in acute cerebral infarction: an 8-year retrospective pilot study. Front Neurol 16:1545631
9. Leppäniemi A, Tolonen M, Tarasconi A, et al (2019) 2019 WSES guidelines for the management of severe acute pancreatitis. World J Emerg Surg 14:27
10. Zhang J, Du JJ, Tang W, et al (2023) CT characteristics of recurrent acute pancreatitis and acute pancreatitis in different stages—a retrospective cross-sectional study. Quant Imaging Med Surg 13(7):4222-4233
11. Enserro DM, Miller A (2025) Improving the Estimation of Prediction Increment Measures in Logistic and Survival Analysis. Cancers (Basel) 17(8):1259

Supplementary Result

Supplementary Result 1. Restricted cubic spline analysis of continuous variables

Before multivariable Cox regression modeling, restricted cubic spline (RCS) regression was applied to assess the linearity assumption between each continuous variable and the log hazard of recurrence. The variables examined included alanine aminotransferase (ALT), Delta-Radscore, VP-Radscore, and NC-3mm-Radscore.

For ALT, the RCS curve displayed a non-monotonic, U-shaped trend; however, the nonlinearity test was not statistically significant (p = 0.118). All other continuous variables demonstrated approximately linear relationships with the log hazard, with p-values for the nonlinearity test > 0.05. To maintain model parsimony and interpretability without sacrificing fit, all continuous variables were included as linear terms in the final multivariable models. Detailed RCS plots for each variable are presented in Supplementary Figure S1.

Supplementary Result 2. Sensitivity analysis of model variable selection

After finalizing the multivariable Cox models, two sensitivity analyses were performed to assess the impact of candidate variable inclusion on model performance and the robustness of selected predictors.

Performance evaluation with low-frequency variable inclusion

After developing the clinical model, we evaluated the effect of adding "exercise"—a variable with low frequency in stability screening (26.7% across 100 bootstrap samples)—on predictive performance. Adding "exercise" to the model, which already included core clinical predictors and radiomics scores, increased the Harrell's C-index in the internal validation cohort from 0.748 (95% CI: 0.671–0.825) to 0.762 (95% CI: 0.689–0.836), yielding an incremental ΔC-index of 0.014. Although this increment slightly exceeded the preset threshold of 0.01, the variable's low selection frequency (26.7%) raised concerns about its robustness and replicability across datasets. Therefore, "exercise" was excluded from the final model. Similarly, the inclusion of other low-frequency variables (e.g., age, sex, body mass index) did not improve the C-index by more than 0.01 in the internal validation cohort, further supporting the parsimony and stability of the current model. These findings suggest that combining stability screening with incremental performance assessment allows for the development of a streamlined model that balances predictive accuracy and robustness.

Rationale for retaining a marginally significant variable

Although "pancreatic enlargement pattern" did not reach conventional statistical significance in the multivariable model (p = 0.061), several considerations supported its retention in the final model. First, the likelihood ratio test provided evidence of improved overall model fit after its inclusion (χ^2 = 3.92, df = 1, p = 0.048). Second, removing this variable resulted in a reduction in the Harrell's C-index in the internal validation cohort from 0.783 to 0.772, corresponding to an incremental ΔC-index of 0.011 (Table S6). Overall, the decision to retain "pancreatic enlargement pattern" was supported by three complementary lines of evidence: (1) its high bootstrap selection frequency during stability screening, (2) improved overall model fit according to the likelihood ratio test (p = 0.048), and (3) a small but consistent decrease in discrimination following its removal. Taken together, these findings supported retaining the variable despite its marginal Wald p-value, as predictor selection in prognostic modeling was based on the overall contribution to model stability, fit, and predictive performance rather than statistical significance from a single multivariable analysis alone.

Supplementary Table

Table S1. Performance of the nnU-Net-based pancreas segmentation model

Fold	Dice	IoU	Recall	Precision
Fold 0	0.853	0.745	0.816	0.907
Fold 1	0.863	0.759	0.857	0.869
Fold 2	0.858	0.753	0.845	0.880
Fold 3	0.873	0.780	0.834	0.905
Fold 4	0.858	0.753	0.845	0.868
Test	0.918	0.853	0.938	0.914

IoU, intersection over union

Table S2. Baseline characteristics of the study cohorts

Variable	Training cohort (n = 272)	Internal validation cohort (n= 117)	External validation cohort (n= 159)	*p*
**Clinical characteristics**				
Age (years)	41.5 (32–53)	40 (33–51)	44 (34–55.3)	0.174
Sex, male	180 (66.2%)	88 (75.2%)	95 (60.9%)	**0.045**
BMI (kg/m²)	26.1 (23.4–29.0)	26.6 (24.2–29.1)	26.0 (22.9–28.7)	0.237
Smoking history	68 (25.0%)	35 (29.9%)	45 (28.3%)	0.551
Alcohol consumption	86 (31.6%)	42 (35.9%)	50 (31.5%)	0.673
Regular exercise	173 (63.6%)	69 (59.0%)	79 (49.7%)	**0.018**
Hypertension	66 (24.3%)	23 (19.7%)	58 (36.5%)	**0.003**
Diabetes mellitus	58 (21.3%)	30 (25.6%)	59 (37.6%)	**0.001**
Etiology				0.091
Hypertriglyceridemia	119 (43.8%)	50 (42.7%)	79 (49.7%)	
Biliary	75 (27.6%)	28 (23.9%)	32 (20.1%)	
Alcoholic	21 (7.7%)	9 (7.7%)	5 (3.1%)	
Mixed	18 (6.6%)	14 (12.0%)	10 (6.3%)	
Idiopathic	39 (14.3%)	16 (13.7%)	33 (20.8%)	
**Vital signs**				
Body temperature(°C)	36.6 (36.4–36.8)	36.5 (36.3–36.8)	36.5 (36.3–36.7)	0.071
Pulse rate(beats/min)	96 (80–110)	98 (86–111)	94 (78–105)	**0.039**
Respiratory rate (breaths/min)	20 (19–21)	20 (19–22)	20 (18–20)	0.149
**Laboratory parameters**				
Serum amylase (U/L)	365 (170–1028)	388 (209–947)	343 (114–920)	0.189
Serum lipase (U/L)	1184(564–2079)	1246 (490–2749)	1713 (642–5042)	**0.021**
Serum calcium (mmol/L)	2.12 (1.96–2.26)	2.11 (1.88–2.24)	2.21 (2.00–2.29)	**0.008**
WBC (×10⁹/L)	14.9 (12.1–18.0)	14.1 (11.5–18.1)	13.8 (10.5–17.0)	0.094
NLR	9.87(5.91–15.68)	8.58 (5.38–13.98)	9.38 (6.71–16.98)	0.304
Peak CRP (mg/L)	166 (99–216)	173 (128–242)	220 (137–302)	**< 0.001**
D-dimer (mg/L)	1.88 (0.86–3.37)	1.71 (0.67–3.54)	1.50 (0.68–2.95)	0.292
Fibrinogen (g/L)	5.42 (3.85–7.11)	5.35 (4.00–6.95)	3.67 (3.08–5.25)	**< 0.001**
ALT (U/L)	34 (21–65)	33 (23–68)	44 (26–99)	**0.013**
Total bilirubin (μmol/L)	18.6 (13.2–27.3)	19.0 (13.5–28.0)	16.2 (12.5–28.4)	0.692
Albumin (g/L)	42.2 (36.5–45.6)	41.0 (37.0–45.3)	40.4 (36.9–43.4)	0.099
LDH (U/L)	291 (216–447)	285 (223–423)	289 (205–478)	0.913
Creatinine (μmol/L)	59.8 (44.0–74.0)	66.0 (52.0–80.0)	57.6 (47.7–69.8)	**0.033**
Urea (mmol/L)	4.80 (3.60–6.09)	5.13 (4.08–6.30)	5.10 (3.79–6.48)	0.367
Triglycerides (mmol/L)	3.80(1.55–12.19)	6.00 (2.03–18.80)	5.43 (1.52–24.40)	**0.04**
Total cholesterol (mmol/L)	6.00 (4.31–8.13)	6.60 (4.65–9.98)	6.07 (4.14–11.32)	0.34
Fasting glucose (mmol/L)	8.15(6.40–11.05)	8.90 (6.77–14.00)	9.22 (6.80–10.98)	0.134
**Severity and CT findings**				
SIRS	145 (53.3%)	68 (58.1%)	77 (48.4%)	0.276
BISAP score				0.529

**Variable**	**Training cohort (n = 272)**	**Internal validation cohort (n= 117)**	**External validation cohort (n= 159)**	***p***
0	48 (17.6%)	15 (12.8%)	37 (23.3%)	
1	102 (37.5%)	46 (39.3%)	61 (38.4%)	
2	103 (37.9%)	47 (40.2%)	52 (32.7%)	
3	18 (6.6%)	9 (7.7%)	9 (5.7%)	
4	1 (0.4%)	0 (0%)	0 (0%)	
Severity				0.941
Mild	3 (1.1%)	1 (0.9%)	1 (0.6%)	
Moderate	132 (48.5%)	55 (47.0%)	83 (52.2%)	
Severe	100 (36.8%)	42 (35.9%)	51 (32.1%)	
Critical	37 (13.6%)	19 (16.2%)	24 (15.1%)	
mCTSI	6 (4–6)	6 (4–6)	4 (2–6)	0.577
Pancreatic morphology (NP)	63 (23.2%)	25 (21.4%)	36 (22.6%)	0.928
Pancreatic enlargement pattern(diffuse)	198 (72.8%)	81 (69.2%)	98 (61.6%)	0.054
Extensive necrosis	3 (1.1%)	3 (2.6%)	11 (6.9%)	**0.003**
Hepatic steatosis	126 (46.3%)	58 (49.6%)	75 (47.2%)	0.841
Pleural effusion	172 (63.2%)	80 (68.4%)	110 (69.2%)	0.379
Ascites	113 (41.5%)	46 (39.3%)	81 (50.9%)	0.09
Onset-to-CT time (days)	7 (5–8)	7 (5–9)	5 (3–7)	**< 0.001**
**Treatment and outcomes**				
Etiology-targeted prevention	178 (65.4%)	83 (71.0%)	111 (69.8%)	0.468
Length of hospital stay (days)	11 (8–16)	12 (8–17)	9 (7–13)	**< 0.001**

Values in bold indicate statistical significance ($p < 0.05$). Group comparisons were performed using the Kruskal–Wallis test for continuous variables and the chi-square test or Fisher's exact test for categorical variables. BMI, body mass index; WBC, white blood cell; NLR, neutrophil-to-lymphocyte ratio; CRP, C-reactive protein; ALT, alanine aminotransferase; LDH, lactate dehydrogenase; SIRS, systemic inflammatory response syndrome; BISAP, bedside index for severity in acute pancreatitis; mCTSI, modified CT severity index; NP, necrotizing pancreatitis

Table S3. Multivariable Cox regression analysis of the clinical model in the training cohort

Variable	β	HR	95% CI	***p***
Onset-to-CT time	0.165	1.179	1.021–1.362	0.025
ALT	-0.767	0.465	0.270–0.800	0.006
Pancreatic enlargement pattern	0.725	2.064	1.158–3.677	0.014
Etiology-targeted prevention	-1.940	0.144	0.088–0.234	<0.001

β, regression coefficient; HR, hazard ratio; CI, confidence interval; ALT, alanine aminotransferase. Variable coding was as follows: pancreatic enlargement pattern (1 = diffuse,

0 = focal) and etiology-targeted prevention (1 = yes, 0 = no). All continuous variables were z-score standardized.

Table S4. Multivariable Cox regression analysis of the radiomics model in the training cohort

Variable	β	HR	95% CI	***p***
Onset-to-CT time	0.059	1.061	0.947–1.189	0.310
Delta-Radscore	0.954	2.596	1.866–3.610	<0.001
VP-Radscore	0.659	1.933	1.377–2.714	<0.001
NC-3mm-Radscore	0.761	2.141	1.186–3.868	0.012

HR, hazard ratio; CI, confidence interval; β, regression coefficient; Delta-Radscore, the radiomics score derived from the percentage change between contrast-enhanced (arterial and portal venous phase) and non-contrast radiomics features; VP-Radscore, the venous-phase radiomics score; and NC-3mm-Radscore, the radiomics score of the peripancreatic region extending 3 mm outward on non-contrast CT.

Table S5. Detailed characteristics and standardized coefficients of selected radiomics features for constructing the NC-3mm, VP, and Delta Radscores

Radscore Type	Selected Radiomics Features	Selection Frequency	Mean	SD	Coefficient (Standardized)
**NC-3mm**	wavelet-HHH_glszm_GrayLevelNonUniformityNormalized	0.62	0.5012	0.00206	0.1876
	wavelet-HHL_glcm_MCC	0.57	0.3061	0.19943	-0.24928
	wavelet-HHL_glcm_MaximumProbability	0.54	0.29282	0.01821	-0.32196
	wavelet-LHH_firstorder_Mean	0.51	0.00164	0.03464	-0.26671
	gradient_firstorder_Kurtosis	0.5	29.35696	40.50904	-0.3121
**VP**	lbp-3D-m2_firstorder_Maximum	0.97	20.12791	0.15272	-0.21758
	wavelet-LHH_glszm_GrayLevelNonUniformityNormalized	0.96	0.52438	0.0327	0.30218
	wavelet-HHL_glszm_GrayLevelVariance	0.93	0.22625	0.03113	-0.26742
	logarithm_firstorder_Kurtosis	0.71	16.74317	23.81576	-0.28299

Radscore Type	Selected Radiomics Features	Selection Frequency	Mean	SD	Coefficient (Standardized)
Delta	exponential_glszm_SizeZoneNonUniformity	0.66	1.01178	0.08659	0.2138
	wavelet-LHH_glcm_JointEntropy	0.61	1.92862	0.00491	-0.20561
	wavelet-HLH_firstorder_Mean	0.6	1.83×10^{-8}	2.81×10^{-6}	-0.23976
	wavelet-HHL_gldm_HighGrayLevelEmphasis	0.57	2.49983	0.00578	0.2542
	del_AN_wavelet-LHH_glcm_ClusterShade	0.65	-1.0565	8.31779	0.21279
	del_VN_wavelet-HHL_glszm_GrayLevelNonUniformityNormalized	0.59	0.03278	0.13899	0.25218
	del_AN_lbp-2D_firstorder_10Percentile	0.57	0.00529	0.10287	-0.30853
	del_VN_log-sigma-3-0-mm-3D_firstorder_Skewness	0.55	2.31218	57.97021	0.16827
	del_VN_wavelet-HLH_firstorder_Kurtosis	0.51	0.23844	0.99047	-0.45536
	del_AN_wavelet-HLH_glszm_GrayLevelNonUniformityNormalized	0.5	0.00748	0.08411	0.2351

Radscore Formulas

The specific Radscores were calculated using standardized feature values via the following formulas:

1. NC-3mm-Radscore Formulation: NC-3mm-Radscore = 0.18760 × (wavelet-HHH_glszm_GrayLevelNonUniformityNormalized) − 0.24928 × (wavelet-HHL_glcm_MCC) − 0.32196 × (wavelet-HHL_glcm_MaximumProbability) − 0.26671 × (wavelet-LHH_firstorder_Mean) − 0.31210 × (gradient_firstorder_Kurtosis)

2. VP-Radscore Formulation: VP-Radscore = −0.21758 × (lbp-3D-m2_firstorder_Maximum) + 0.30218 × (wavelet-LHH_glszm_GrayLevelNonUniformityNormalized) − 0.26742 × (wavelet-HHL_glszm_GrayLevelVariance) − 0.28299 × (logarithm_firstorder_Kurtosis) + 0.21380 × (exponential_glszm_SizeZoneNonUniformity) − 0.20561 × (wavelet-LHH_glcm_JointEntropy) − 0.23976 × (wavelet-HLH_firstorder_Mean) + 0.25420 × (wavelet-HHL_gldm_HighGrayLevelEmphasis)

3. Delta-Radscore Formulation: Delta-Radscore = 0.21279 × (del_AN_wavelet-LHH_glcm_ClusterShade) + 0.25218 × (del_VN_wavelet-HHL_glszm_GrayLevelNonUniformityNormalized) − 0.30853 × (del_AN_lbp-2D_firstorder_10Percentile) + 0.16827 × (del_VN_log-sigma-3-0-mm-3D_firstorder_Skewness) − 0.45536 × (del_VN_wavelet-HLH_firstorder_Kurtosis) + 0.23510 × (del_AN_wavelet-HLH_glszm_GrayLevelNonUniformityNormalized)

The prefixes "del_AN" and "del_VN" denote the relative change of a given radiomics feature between the contrast-enhanced phase (arterial and portal venous phase, respectively) and the non-contrast (NC) phase. This relative change is calculated as: (Feature Value_enhanced − Feature Value_NC) / Feature Value_NC. SD, standard deviation; NC, non-contrast; VP, venous phase.

Table S6. Impact of the pancreatic enlargement pattern on model discriminatory performance

Dataset	Full model C-index (95% CI)	Reduced model C-index (95% CI)	ΔC-index (95% CI)
Training cohort	0.831 (0.791–0.872)	0.822 (0.780–0.863)	0.009 (−0.001, 0.023)[1]
Internal validation cohort	0.783 (0.699–0.867)	0.772 (0.686–0.862)	0.011 (−0.011, 0.029)[1]

C-index, concordance index; ΔC-index, difference in C-index between the full model (including the pancreatic enlargement pattern) and the reduced model (excluding this variable).

[1] Point estimates derived from the original data; 95% CIs derived from 1000 bootstrap resamples using the fixed-coefficient method (percentile intervals).

Table S7. Comparison of model fit statistics in the training cohort

Model	–2 log-likelihood	AIC	Likelihood ratio test (vs. combined model)
Clinical model	378.56	765.1	$\chi^2 = 43.30$, df = 3, $p < 0.001$
Radiomics model	392.61	793.2	$\chi^2 = 71.40$, df = 3, $p < 0.001$
Combined model	356.91	727.8	Reference

Likelihood ratio tests compared each reduced model (clinical or radiomics) against the full combined model to assess whether adding the respective set of variables significantly improved model fit. AIC, Akaike Information Criterion.

Table S8. Comparison of predictive performance between single-phase and multiphasic radiomics models across study cohorts (adjusted for onset-to-CT time)

Radiomics Model	Training cohort C-index (95% CI)	Internal validation cohort C-index (95% CI)	External validation cohort C-index (95% CI)
**Non-contrast (NC) phase only**	0.554 (0.485, 0.624)	0.543 (0.444, 0.641)	NA
**NC-3mm**	0.550 (0.484, 0.617)	0.607 (0.506, 0.708)	NA
**NC-5mm**	0.571 (0.504, 0.639)	0.571 (0.483, 0.659)	NA
**Arterial phase (AP) only**	0.604 (0.537, 0.670)	0.596 (0.501, 0.690)	NA
**Venous phase (VP) only**	0.679 (0.619, 0.739)	0.618 (0.539, 0.698)	NA
**Delta**	0.637 (0.566, 0.708)	0.623 (0.562, 0.684)	NA
**Multiphasic integration (NC-3mm,VP and Delta)**	0.723 (0.669, 0.778)	0.647 (0.538, 0.757)	0.619 (0.536, 0.703)

NA, not assessed. Single-phase models were not subjected to external validation due to their suboptimal discriminative performance during the development and internal validation phases. To ensure a fair comparison and mitigate temporal bias, the interval from symptom onset to CT examination (onset-to-CT time) was mandatorily included as an adjustment covariate when calculating the C-index for all candidate models. C-index, concordance index; CI, confidence interval; Delta refers to the relative change rate of radiomic feature values between the contrast-

enhanced phases (arterial or venous) and the non-contrast phase.

Table S9. Time-dependent AUC of prediction models at different follow-up time points

Follow-up (months)	Model	AUC (95% CI)		
		Training cohort	Internal validation cohort	External validation cohort
12	Clinical model	0.807 (0.733-0.882)	0.784 (0.660-0.908)	0.781 (0.671-0.891)
	Radiomics model	0.725 (0.644-0.807)	0.721 (0.528-0.914)	0.579 (0.454-0.704)
	Combined model	0.877 (0.828-0.925)	0.829 (0.691-0.968)	0.782 (0.677-0.888)
18	Clinical model	0.767 (0.695-0.838)	0.805 (0.697-0.913)	0.745 (0.645-0.845)
	Radiomics model	0.740 (0.669-0.810)	0.655 (0.482-0.828)	0.637 (0.526-0.747)
	Combined model	0.838 (0.781-0.895)	0.822 (0.699-0.945)	0.798 (0.709-0.886)
24	Clinical model	0.761 (0.687-0.835)	0.764 (0.652-0.877)	0.724 (0.627-0.820)
	Radiomics model	0.727 (0.652-0.802)	0.670 (0.521-0.819)	0.640 (0.530-0.750)
	Combined model	0.835 (0.775-0.894)	0.802 (0.696-0.908)	0.785 (0.699-0.872)
30	Clinical model	0.777 (0.704-0.849)	0.756 (0.641-0.871)	0.761 (0.668-0.854)
	Radiomics model	0.738 (0.664-0.812)	0.672 (0.519-0.824)	0.649 (0.542-0.755)
	Combined model	0.845 (0.786-0.903)	0.798 (0.690-0.907)	0.809 (0.724-0.893)
36	Clinical model	0.804 (0.734-0.874)	0.754 (0.638-0.871)	0.769 (0.662-0.876)
	Radiomics model	0.766 (0.693-0.840)	0.606 (0.462-0.751)	0.629 (0.507-0.750)
	Combined model	0.871 (0.815-0.927)	0.773 (0.660-0.886)	0.804 (0.709-0.899)
42	Clinical model	0.848 (0.787-0.909)	0.741 (0.620-0.863)	0.698 (0.559-0.837)
	Radiomics model	0.769 (0.694-0.845)	0.626 (0.488-0.763)	0.651 (0.511-0.790)
	Combined model	0.903 (0.855-0.952)	0.786 (0.675-0.897)	0.769 (0.646-0.891)
48	Clinical model	0.859 (0.799-0.918)	0.743 (0.617-0.870)	0.700 (0.524-0.876)
	Radiomics model	0.753 (0.672-0.834)	0.624 (0.482-0.766)	0.638 (0.467-0.809)
	Combined model	0.903 (0.852-0.954)	0.776 (0.655-0.897)	0.686 (0.502-0.870)
54	Clinical model	0.877 (0.818-0.936)	0.724 (0.591-0.858)	0.679 (0.375-0.973)
	Radiomics model	0.734 (0.645-0.823)	0.637 (0.493-0.781)	0.598 (0.309-0.905)
	Combined model	0.905 (0.849-0.961)	0.760 (0.633-0.887)	0.697 (0.387-0.997)

AUC, area under the curve; CI, confidence interval

Supplementary Figure

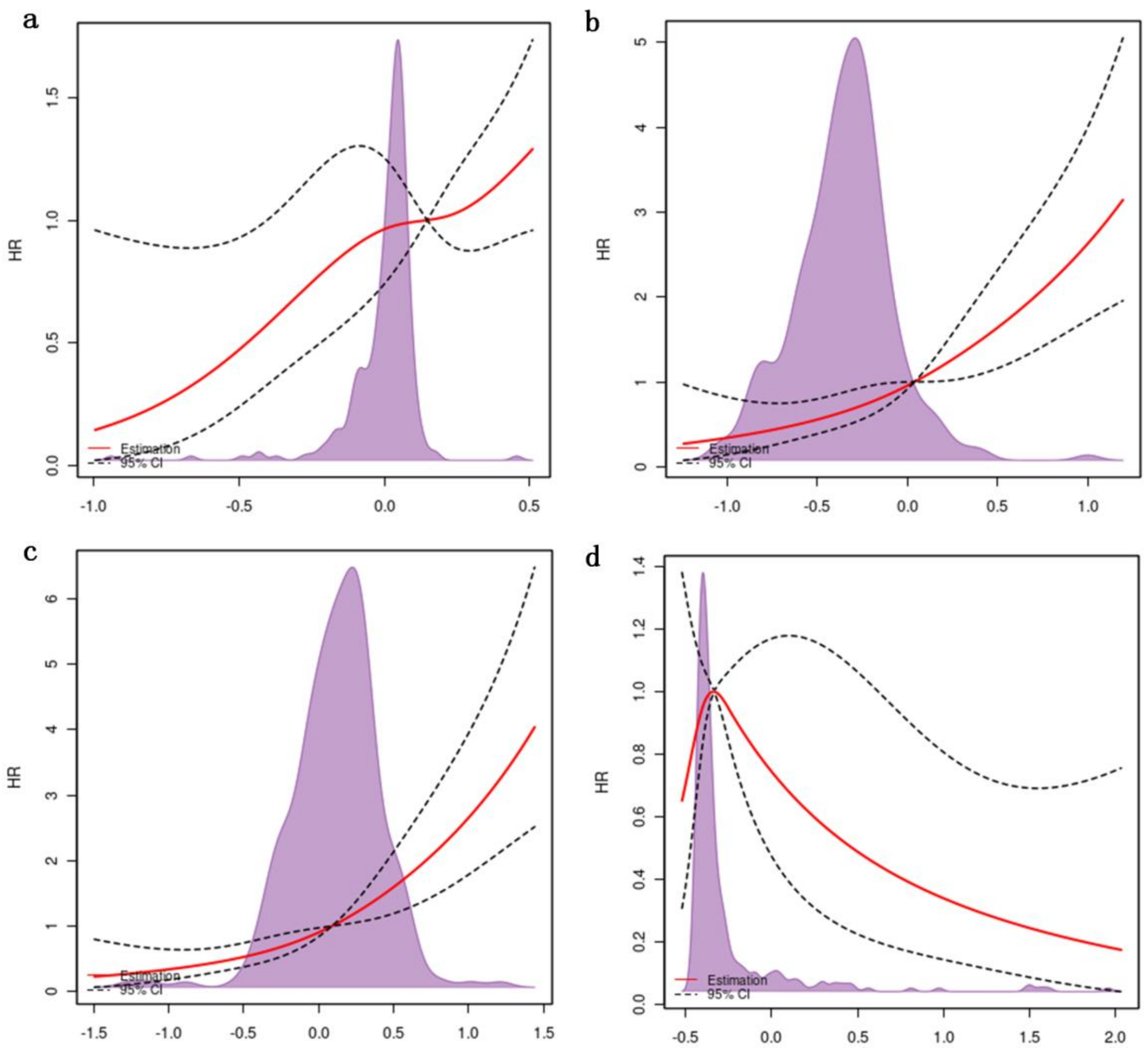

Figure S1. Restricted cubic spline (RCS) analyses of individual continuous predictors in the training cohort (n = 272). **(a)** Association between the non-contrast phase radiomics score (NC-3mm-Radscore) and the log hazard ratio (log[HR]) of acute pancreatitis (AP) recurrence; **(b)** Aassociation between the venous phase radiomics score (VP-Radscore) and log(HR); **(c)** Aassociation between the pancreatic parenchymal phase radiomics score (Delta-Radscore) and log(HR); **(d)** Association between alanine aminotransferase (ALT) levels and log(HR). The x-axis represents the standardized value of each predictor; the y-axis represents the log hazard ratio for recurrence. The solid line indicates the fitted restricted cubic spline curve, with dashed lines representing the 95% confidence interval. The horizontal dashed line at log(HR) = 0 (HR = 1) serves as the reference. The *p*-value for nonlinearity was derived from the likelihood ratio test comparing the model with the RCS term against a linear model.

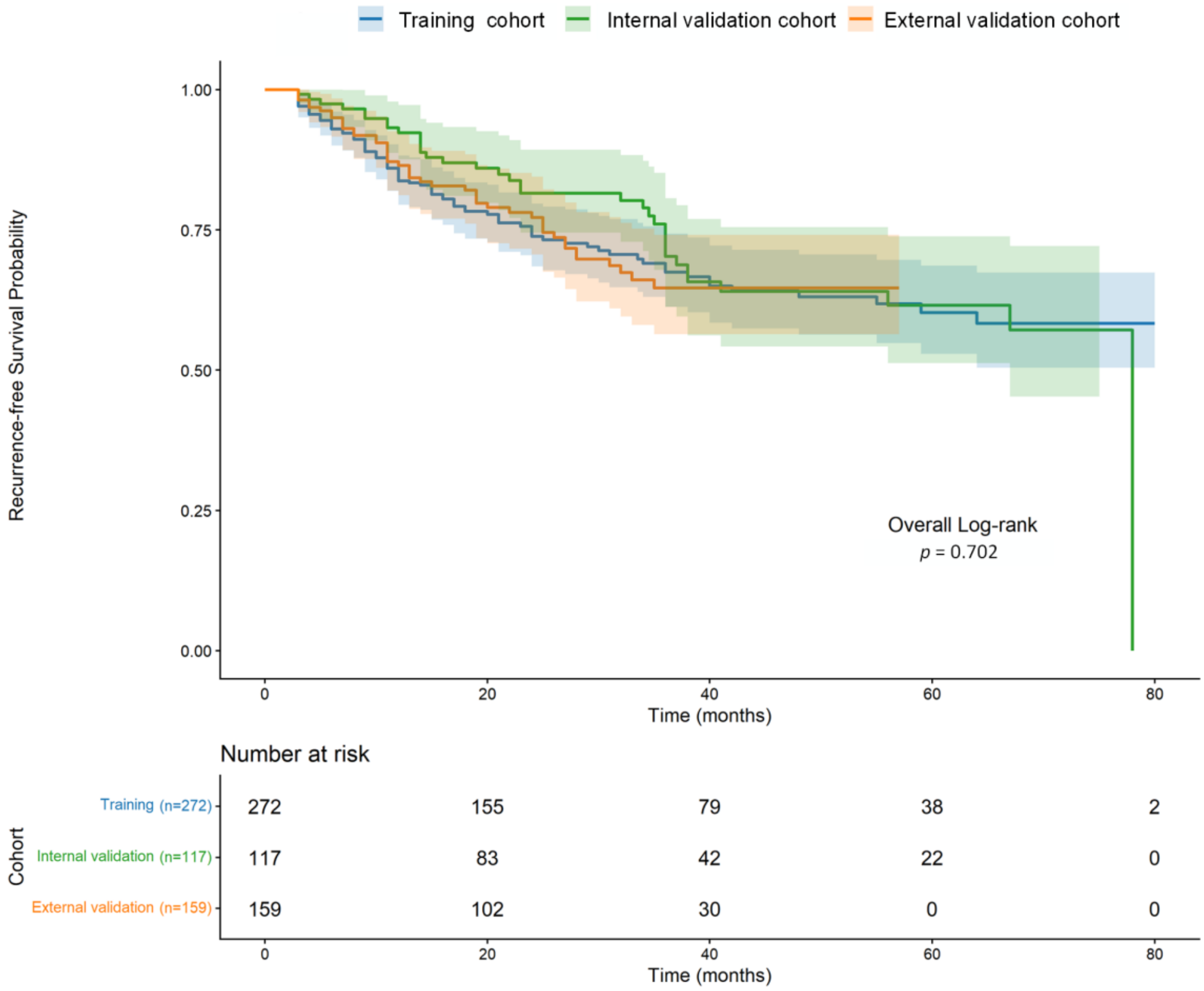

Figure S2. Baseline recurrence-free survival across the three study cohorts. Kaplan–Meier curves depicting recurrence-free survival for patients in the training, internal validation, and external validation cohorts prior to the application of any prediction model. Survival distributions were compared using the log-rank test (p = 0.702).

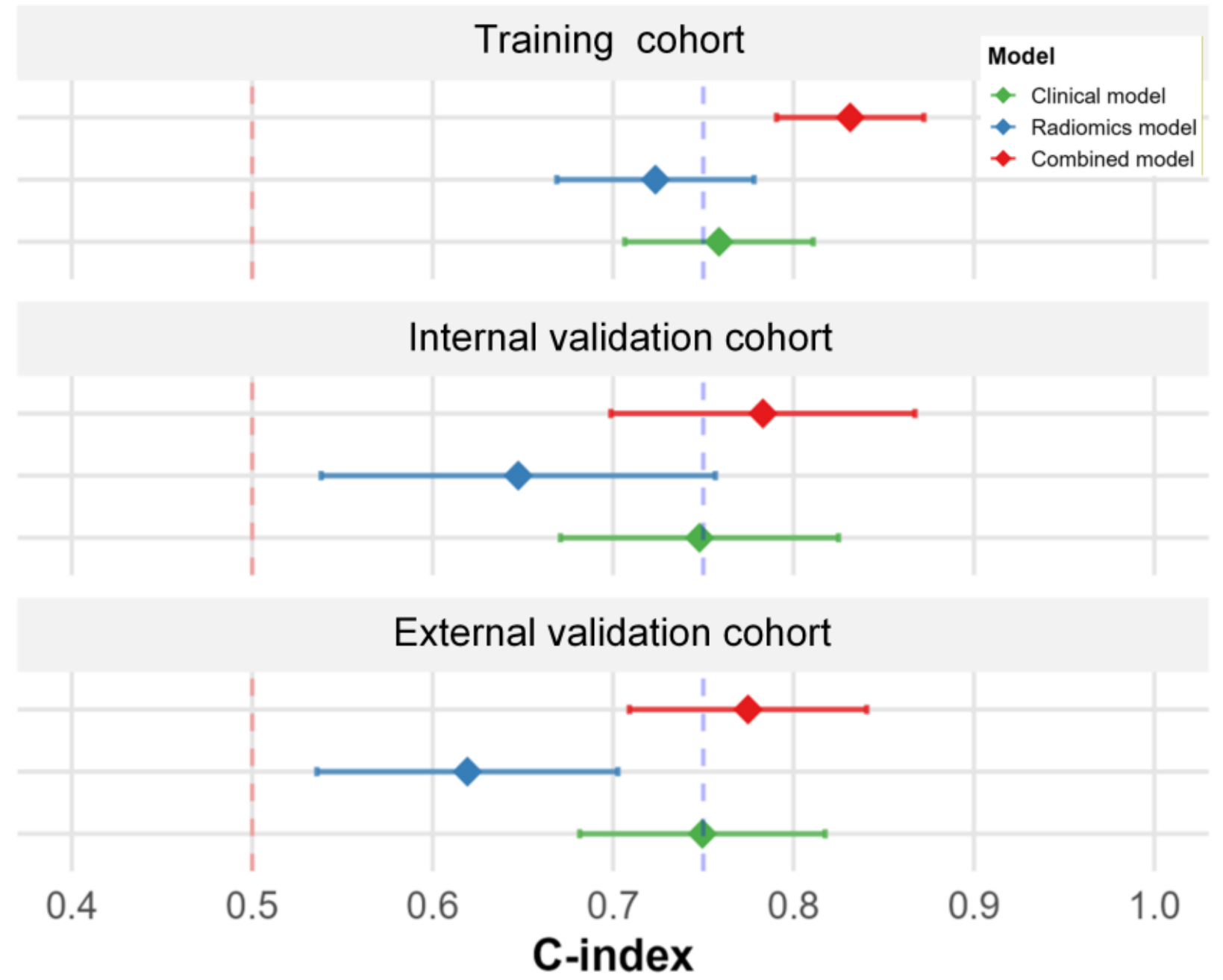

Figure S3. Forest plot of the C-index for each prediction model across the three study cohorts.The plot displays/shows point estimates (squares) with 95% confidence intervals (horizontal lines) for the clinical (green), radiomics (blue), and combined (red) models in the training, internal validation, and external validation cohorts. The vertical dashed line at C-index = 0.5 represents the discrimination performance of random chance.

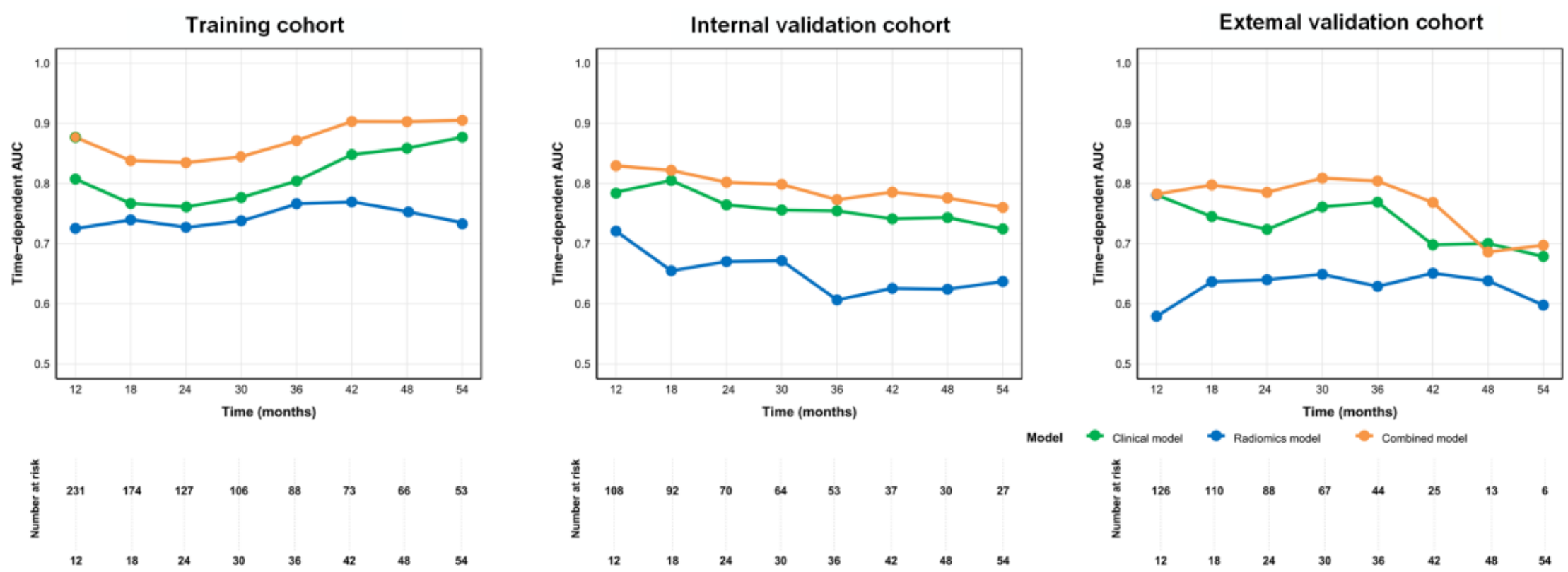

Figure S4. Time-dependent AUC for each prediction model across the three study cohorts. Time-dependent area under the curve (AUC) for the clinical, radiomics, and combined models in the training, internal validation, and external validation cohorts at 12, 18, 24, 30, 36, 42, 48, and 54 months. The number of patients at risk at each time point is shown below the x-axis.

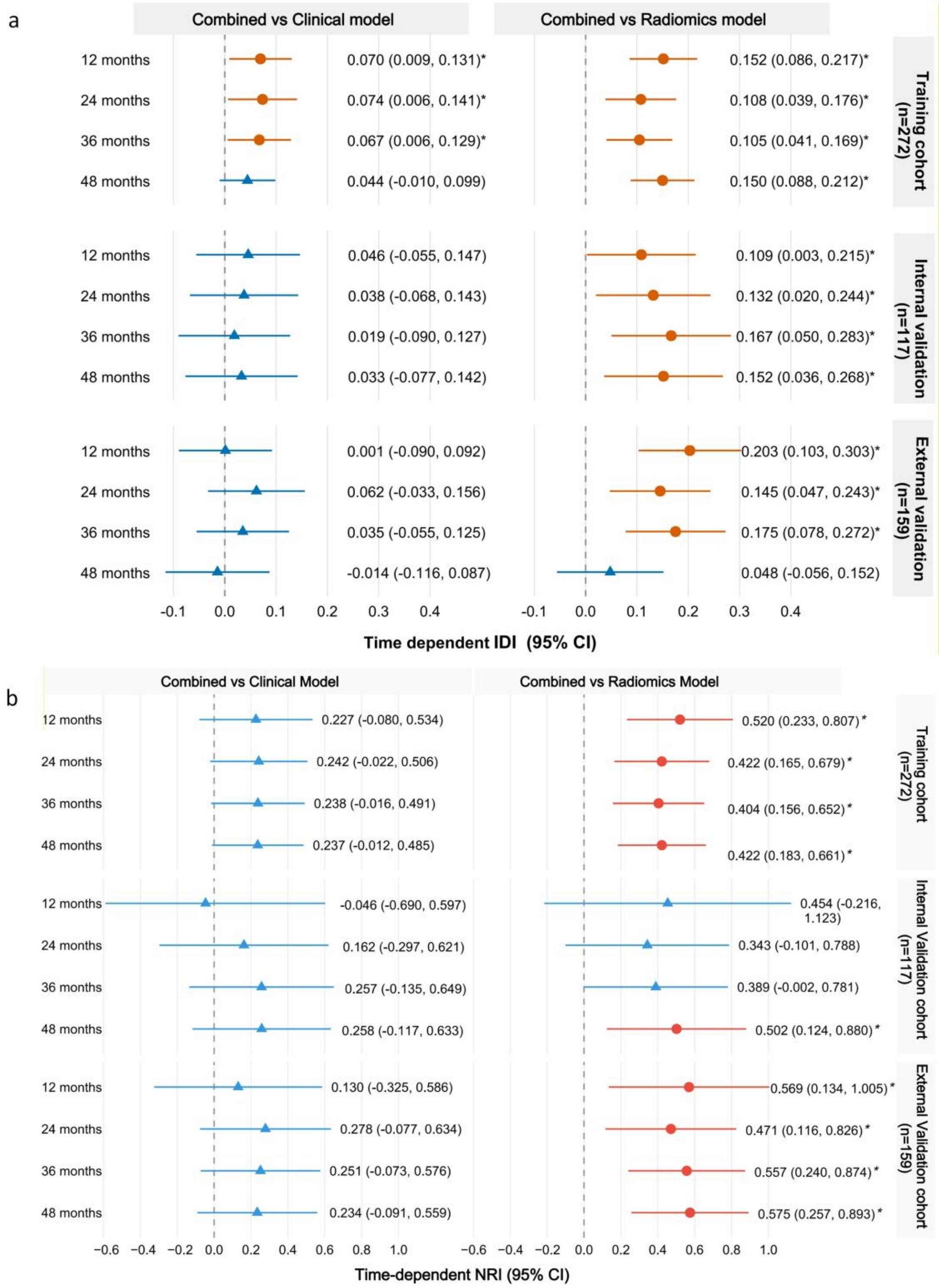

Figure S5. Incremental value of the combined model. **(a)** Time-dependent integrated discrimination improvement (IDI) for the combined model versus the clinical model (left) and versus the radiomics model (right) in the training, internal validation, and external validation cohorts. Error bars represent 95% confidence intervals (CIs). Solid orange circles and asterisks indicate statistically significant improvement (95% CI excluding zero); solid blue triangles indicate non-significant differences.**(b)** Time-dependent net reclassification improvement (NRI) for the combined model versus the clinical model (left) and versus the

radiomics model (right) in the three cohorts. Symbols are as in (a). The vertical dashed line marks the null value (0).

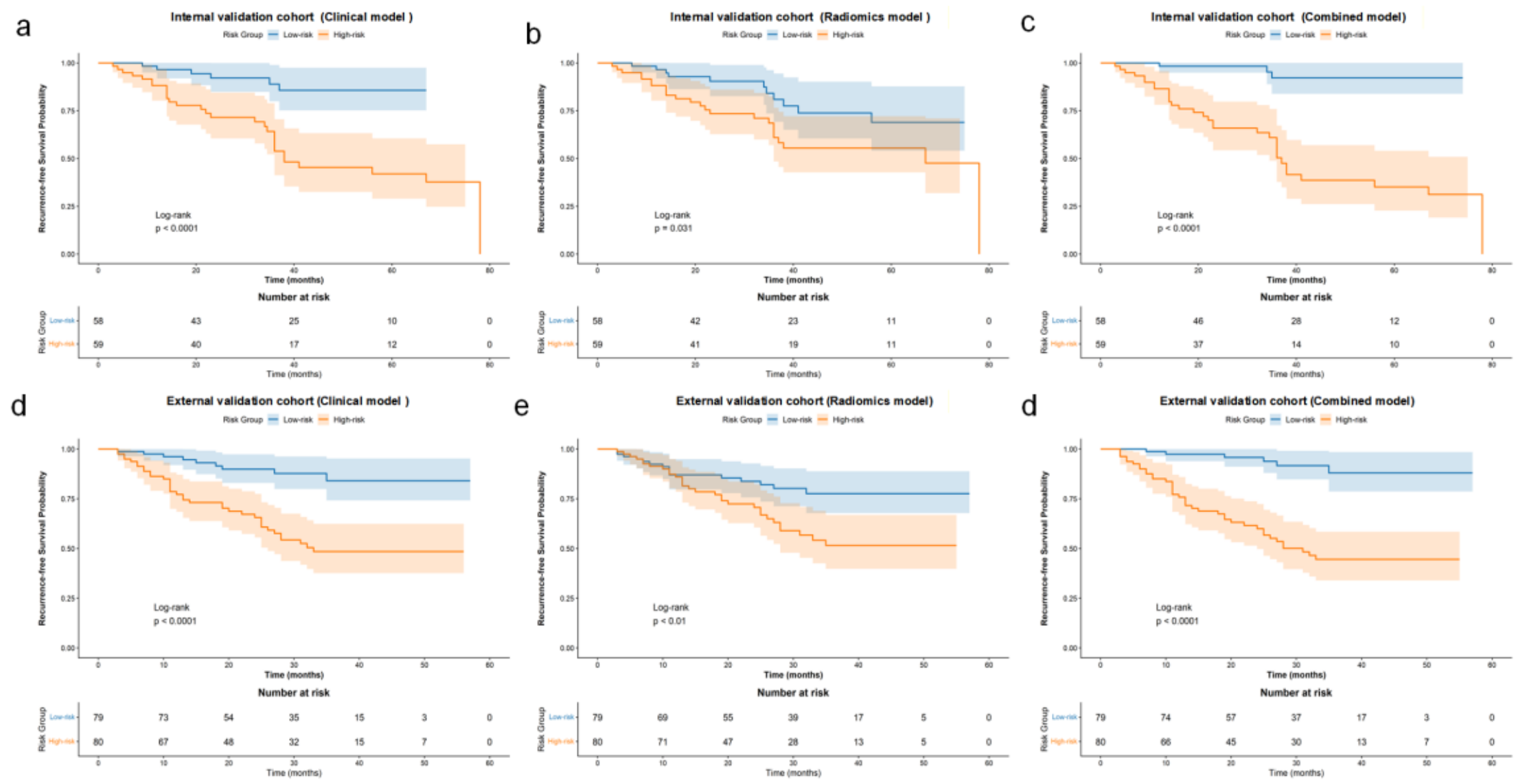

Figure S6. Direct comparison of risk stratification by the three models in the validation cohorts.

(a–c) Kaplan–Meier curves for recurrence-free survival in the internal validation cohort, stratified by the clinical model **(a)**, the radiomics model **(b)**, and the combined model **(c)** using the respective median risk score from the training cohort as the cutoff. **(d–f)** Corresponding Kaplan–Meier curves in the external validation cohort for the clinical model **(d)**, the radiomics model **(e)**, and the combined model **(f)**, applying the same cutoff values derived from the training cohort. The combined model consistently achieved the most effective risk stratification, with the widest separation between high- and low-risk groups and the lowest log-rank *p*-values in both validation cohorts.

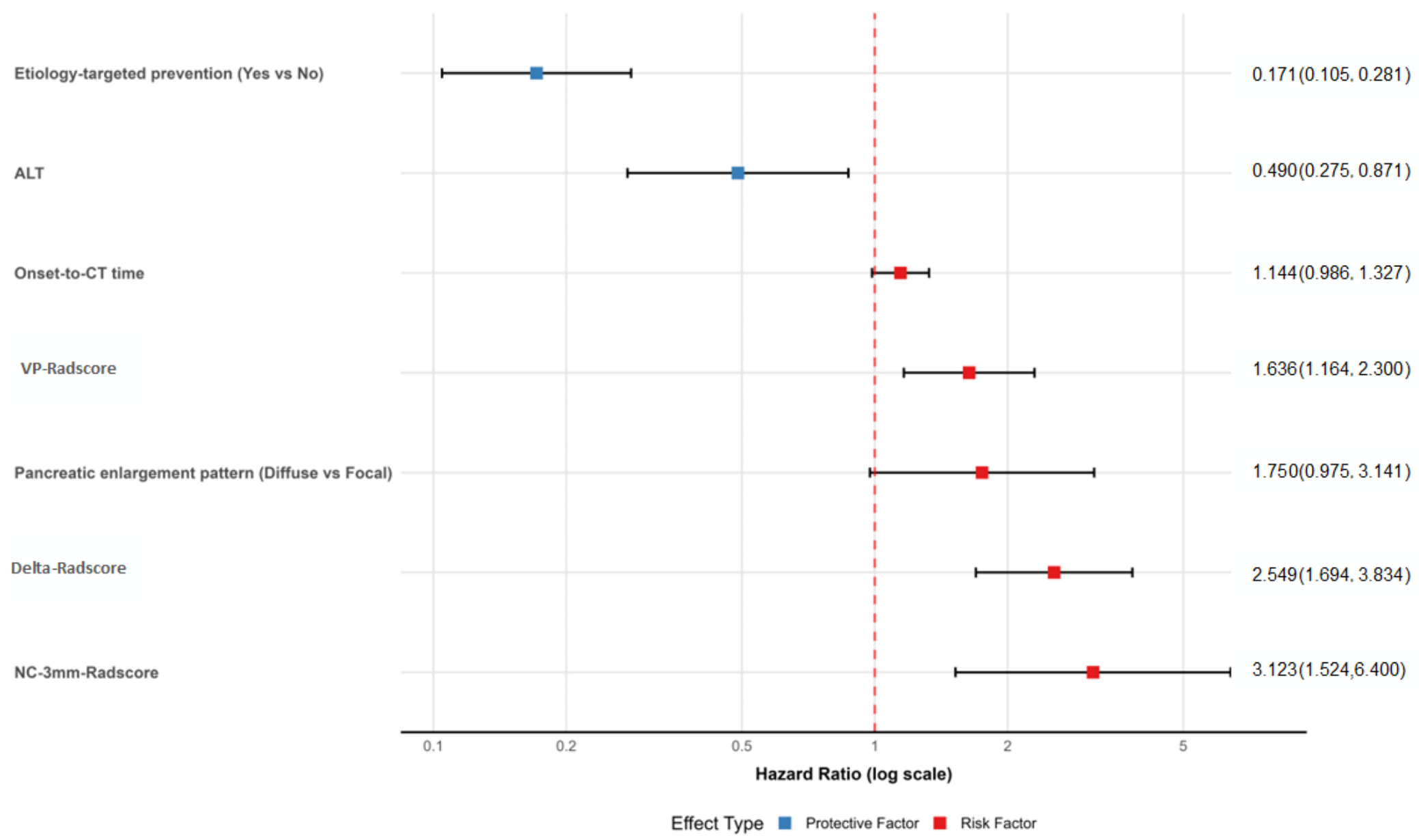

Figure S7. Forest plot of multivariable Cox regression analysis in training cohort (n = 272). Forest plot displaying the hazard ratios (HRs) and 95% confidence intervals (CIs) for independent predictors of acute pancreatitis recurrence derived from the multivariable Cox proportional hazards model. Blue squares indicate protective factors (HR < 1); red squares indicate risk factors (HR > 1).

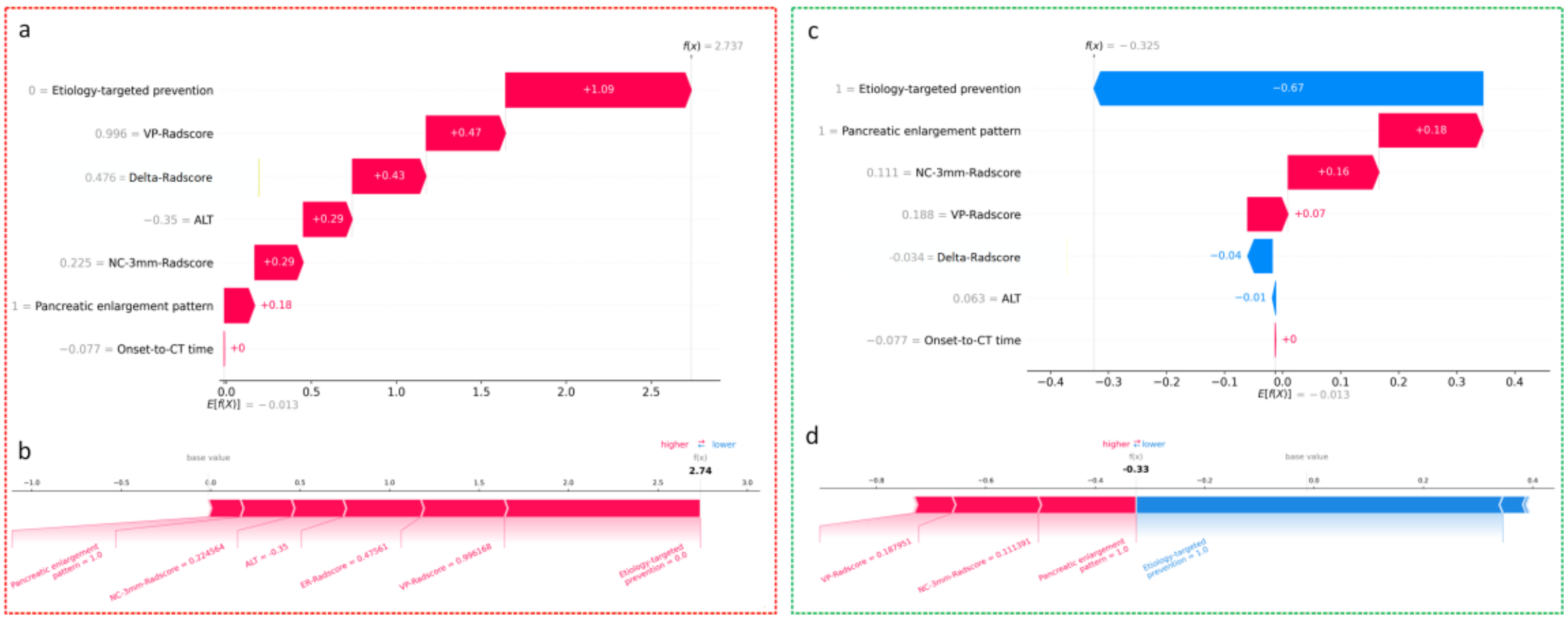

Figure S8. SHAP waterfall plots and force plots illustrating the feature contribution to Patient 1 (high-risk) and Patient 2 (low-risk). **(a, b)** For Patient 1 (CPI = 1.991), the waterfall plot **(a)** quantifies the positive SHAP contributions of key risk factors (elevated NC-3mm-Radscore, VP-Radscore, and diffuse pancreatic enlargement pattern) that drive the prediction significantly above the cohort baseline. The force plot **(b)** visually confirms that the absence of etiology-targeted prevention and high radiomics scores are the dominant drivers of the high-risk classification. **(c, d)** For Patient 2 (CPI = –1.073), the waterfall plot **(c)** shows prominent negative contributions from etiology-targeted prevention (value = 1) that offset the minor positive effects of individual radiomics features. The force plot **(d)** demonstrates that this strong protective effect shifts the final prediction substantially below the cohort baseline, consistent with the 48-month recurrence-free follow-up outcome. All continuous variables were z-score normalized prior to model input.
